# Can trade credit rejuvenate Islamic banking?

**DOI:** 10.1007/s11156-022-01092-6

**Published:** 2022-09-15

**Authors:** Wahyu Jatmiko, M. Shahid Ebrahim, Abdullah Iqbal, Rafal M. Wojakowski

**Affiliations:** 1 Faculty of Economics and Business, University of Indonesia, Depok, West Java 16424 Indonesia; 2 Durham University Business School, University of Durham, Mill Hill Lane, Durham, DH1 3LB UK; 3 Kent Business School, University of Kent, Medway Building, Chatham, ME4 4AG UK; 4 Surrey Business School, University of Surrey, Guildford, GU2 7XH UK

**Keywords:** Crisis, Islamic banking, Mark-up (Murabaha) financing, Trade credit, Universal banking, G21, G32, Z12

## Abstract

This study proposes a renewal of the contemporary Islamic banking Murabaha financing model as it aggravates financial fragility with waning economic efficiency. We adapt the working capital framework of successful US companies like Amazon and Walmart and model an innovative Murabaha facility as trade credit within the *real sector* of the economy. We then test its robustness in a range of simulation tests. Our approach is novel and stands in contrast to the familiar *financial sector* fixed-income facilities, characteristic of Western economies, stealthily mimicked as mark-up (interest rate based) Murabaha by Islamic banks. We argue that this is neither appropriate nor effective for Islamic economies, making them fragile under monetary pressures in crises like the current coronavirus and energy ones. Our simulation results indicate that the trade credit Murabaha not only transforms debt into a risk-sharing one but also offers more competitive financing rates, reduces systemic risk, and improves financial stability. Furthermore, our results imply that the trade credit Murabaha can increase the efficiency of Islamic financial systems and make them more resilient to shocks. Consequently, this paper discusses the integration of our novel Murabaha within a recreated architecture of Universal Banking. As an implication, this should promote business activity and contribute to global growth. Finally, we recommend how to deploy our novel Murabaha based on trade credit (as opposed to the currently deployed fixed-income-mimicked Murabaha) to alleviate twin agency debt costs (risk shifting, underinvestment) and solve the ownership transfer problem of modern Islamic banking.

## Introduction

*General dearth of academic work on Islamic finance stands in contrast with the increasing importance that Islamic banking has in many Muslim countries in Asia and in Africa*.

(Thorsten Beck, Asli Demirgüç-Kunt, and Ouarda Merrouche, 2013, p. 434)

**Fig. 1:**
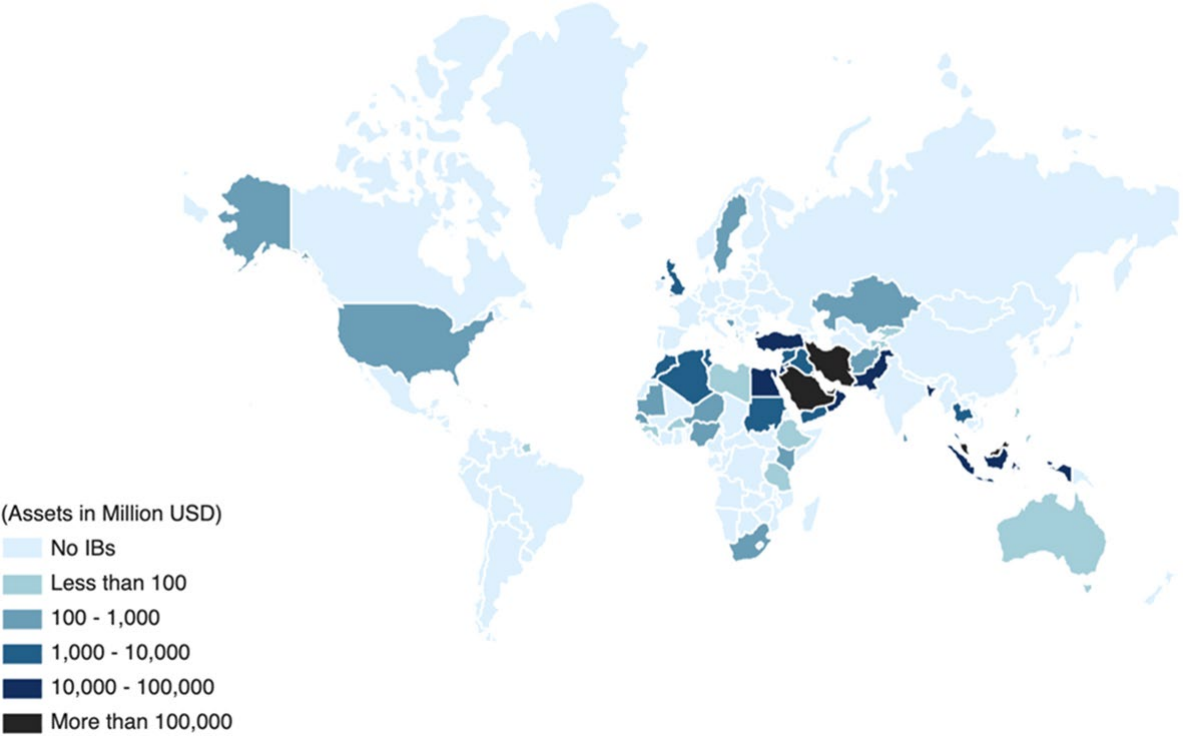
Geography of Islamic banks by total assets. *Note*: This figure exhibits the spread of Islamic Banks’ (IBs’) total assets worldwide as of December 31, 2020. This is based on the latest data available from Datastream, as of January 20, 2022

Islamic banks (IBs—hereafter) finance activities of a wide range of businesses and have experienced exponential growth over the last decade (Abuzayed et al. 2018). For example, Datastream reports that the total assets of IBs, which were US$947 billion in 2008, have grown by more than 148.05% to US$2.349 trillion by the end of 2020. Their total national banking assets proportion is also significant. For example, IBs’ market share exceeds 15% of their respective countries’ total banking assets in at least 15 jurisdictions (IFSB 2021).^1^ More importantly, they not only operate in the majority Muslim countries but also in the minority ones, such as the United Kingdom, Cyprus, the USA, Australia, Thailand, and South Africa (see Fig. 1). In addition, their services are not exclusive to Muslims only. In the UK, for instance, the demographics of IBs’ consumers show that they are primarily non-Muslims (Moore 2011; The Economist 2018; Firdaus 2019).

IBs are value-oriented financial institutions whose business model stems from Islamic jurisprudence (Mergaliyev et al. 2021). In essence, they are supposed to avoid interest-bearing transactions (*ribā*) and those with excessive risk or legally ambiguous (*gharar*) financial contracts. At the onset of the Islamic banking industry, in the 1960s and 1970s, its advocates proposed the two-tier structure involving the medieval quasi-equity profit-sharing facility. That is, on the asset side, the Islamic bank enters into a quasi-equity-based financial contract and links the compensation of its depositors to the performance of its asset side. However, in reality, the practice deviates from this concept for many reasons, including agency issues, competition with conventional banks, complexity in regulation, and lack of human capital (Aggarwal and Yousef 2000). IBs, instead, offer a mark-up facility termed the ‘Murabaha to the purchase order’ (also known as the banking or contemporary Murabaha), whereby they purchase a pre-ordered tangible asset and sell it on a profit margin.^2^ This facility accounts for most of the financing employed by IBs (Baele et al. 2014).

The banking Murabaha is, however, criticized as an ineffective replication of a debt facility.^3^ This operational inefficiency results from the complex set-ups put in place to make the transactions conform to Islamic law (El-Gamal 2006; Gözübüyük et al. 2020).Such a contract mainly stems from two substantial drawbacks, namely (i) the fictitious transfer of ownership and (ii) the use of a fragile debt contract (instead of a resilient risk-sharing one) (Khan 2010).^4^ As the banking Murabaha is the backbone of the IB industry, improving its practice can significantly contribute to “rejuvenating” Islamic Banking.

The inefficiency of IBs is of utmost interest to academics, practitioners, and policymakers for the following reasons. First, IBs are interconnected with the global banking network, allowing them to transmit systemic risks^5^ to the international financial system (Alandejani et al. 2017; Chakroun and Gallali 2017).^6^ Second, previous studies have consistently evidenced the poor risk management of large IBs, leading to even higher contagion risk to the economy (Čihák and Hesse 2010; Abedifar et al. 2013). This is especially true in the current economic environment, though not specific to IBs, where the sudden suspension of business activity designed to arrest coronavirus spread has led to an acute decline in global growth since the Great Depression. This crisis comes after years of lax financial conditions that encouraged businesses to lever up. The extraordinary increase in debt by businesses greater than their earnings growth and cash balances has aggravated their risk of default, thereby threatening the well-being and stability of the global banking system (see Kashkari 2020). Thus, rejuvenating the banking Murabaha as IBs’ most employed contract is the need of the hour. This is because replacing a fragile long-term debt facility with a short-term risk-sharing one has the potential to alleviate the burdensome loan obligations on businesses and help them expand their activities to lead to a global economic recovery.

Extant research on improving the rudimentary Islamic banking system is scarce. They are mainly empirical in nature and focus on contrasting IBs with their conventional counterparts (see Abedifar et al. 2013; Beck et al. 2013; Alandejani et al. 2017; Elnahass et al. 2020; Trinh et al. 2021). Our paper is a unique theoretical study focusing on this research gap and constructively critiquing the current practice of Islamic banking by addressing the following question. *Can the Murabaha financing offered by IBs be improved upon by the disruptive technology conferred by business trade credit* (TC—hereafter)?^7^

Our study intuitively resorts to TC (i.e., financing of business inventories with accounts payables), which serves as an integral element of short-term financing by suppliers of enterprises in addition to bank credit. This source of corporate funding is widely used by not only financially constrained but also investment-grade companies (Murfin and Njoroge 2015). TC can endow efficiency to businesses, revitalizing society and ultimately helping it progress economically (Ge and Qiu 2007).^8^ An excellent example of this premise is that of Amazon Inc., whose shares have phenomenally skyrocketed more than 80,000% after 2 decades of listing on the stock exchange. The retailing giant’s unique and disruptive business model employs a substantial amount of account payable period, estimated to be around 80 days. This bestows it with an overwhelming cash float for investment in profitable projects (Powell 2018). This is not an isolated case as Walmart too uses TC more than bank loans. In 2019, TC for Walmart was 14.5 times the capital amount invested by shareholders (Walmart 2020). Amazon’s and Walmart’s enormous growth are ideal examples of how effective TC is crucial to not only its success but also that of the USA.^9^

TC can potentially redeem the inherent weaknesses of the contemporary Murabaha (i.e., fictitious transfer of ownership and fragile debt-based), thereby enhancing the efficiency of the financial system (Blejer 2006). We argue that the problems mentioned above arise from the inefficiency of conducting the mark-up ‘sale’ transaction in the financial sector. The former (i.e., fictitious transfer of ownership), where IBs sell something that is not in their possession, stems from the banking regulatory restriction on holding real assets (inventories) for business purposes (Grais and Pellegrini 2006). The latter (fragile debt-based) stems from the disconnect between the payoffs of Murabaha banking and the business’s underlying performance in the *real* sector of the economy. This is inevitable as IBs compete in the highly efficient financial sector (El-Gamal 2006). This forces the Islamic Interbank Benchmark Rate (including the Murabaha rate), a daily average of rates contributed by 18 international IBs, to converge to the ongoing market (such as LIBOR) interest rate (Azad et al. 2018). Unlike the banking Murabaha, the mark-up sale facility in the medieval Islamic era incorporated the concept of contemporary TC. Despite the link between the two facilities, IBs have treated the issue of TC (which should be in the *real* sector of the economy) as a substitute for bank credit (which is in the *financial* sector) without deliberating on the nonfinancial reasons behind this issue.

We, therefore, theoretically structure the mark-up credit sale facility in its original (i.e., historical) context, that is, providing TC in accordance with the economic intuition of facilitating trade.^10^ This is because Murabaha in early Islam was employed to increase the demand for goods sold on credit and to complete the markets (Sen 1998). Our approach involves relating this facility to the well-known TC literature (Dass et al. 2015). We then price the TC-Murabaha (i.e., classic Murabaha employed in the *real* sector of the economy) consistent with the objectives of Islamic law (*Maqāsid Al-Sharī’ah*).^11^ Finally, we propose a simple yet meaningful and practicable model and test it using numerical simulations to show the economic efficiency of TC-Murabaha in contrast with the contemporary IB mark-up.^12^ This is conducted by extending the Rashid and Mitra (1999) (R&M) analysis to numerically illustrate the Murabaha discount rate as being lower than interest rates in periods of monetary tightening or financial crisis when banks curtail credit to businesses.^13^

Our results extend not only the research on IBs but also contribute to the broad business working capital (i.e., TC) literature. This is because IBs in our framework act like factors in collecting payments on account receivables. Consistent with Nilsen (2002), Ge and Qiu (2007), Fabbri and Menichini (2010), Klapper and Randall (2011), Chod (2017), Sautner and Vladimirov (2018), and Yang and Birge (2018), we also uncover that retailers (borrowers) (regardless of their sizes and financial constraints) prefer TC (TC-Murabaha) over bank financing (Murabaha banking), especially during the distress periods (such as the current coronavirus one) for financial (cost) and liquidation advantages. It is, however, contrary to Ng et al. (1999), Cuñat (2007), and Klapper et al. (2012), who document no empirical evidence for financial, price discrimination, and liquidity advantages of TC. Instead, they attribute the utilization of TC to the product quality motive.

Our findings also provide valuable policy-related implications for the debate regarding the most appropriate banking system in the post-coronavirus era. We argue that a modified universal banking architecture offers a more efficient developmental sphere for IBs. We amalgamate our results into a universal Islamic banking (or financial conglomerate) architecture by employing: (i) the Japanese concept of *Keiretsu* (Miwa and Ramseyer 2002; Santos and Rumble 2006; Sueyoshi et al. 2010)^14^; and (ii) an altered universal banking concept commonly observed in some European countries (Neuhann and Saidi 2018).^15^ This improved financial structure has the following advantages. First, it benefits from economies of scale and scope. Second, it enhances corporate governance. For instance, Kim and Limpaphayom (1998, p. 37) document “the existence of a Keiretsu two-tier corporate governance system. In the first stage, the unique corporate cross-shareholding allows mutual monitoring under normal circumstances. In the second stage, when firms get into financial trouble, Keiretsu financial institutions assume control by reducing debt levels.” Third, it mitigates systemic risk and, thus, financial fragility. Finally, it advances the underlying businesses’ supply chain and inventory management. These results are coherent with those of Berglöf and Perotti (1994), Petersen and Rajan (1995), Berlin et al. (1996), Gorton and Schmid (2000), and Chod (2017).^16^

The rest of the paper is organized as follows. First, we discuss our institutional framework by integrating the Islamic value system with the TC literature in Sect. 2. Second, we develop our pricing theory of the TC-based Murabaha facility in Sect. 3. To this end, we employ motivations from vendor financing, and, as a result, we substantially contribute to the business working capital literature. We then numerically test our theory and assimilate our results to realize a universal Islamic banking architecture in Sect. 4. Finally, we conclude our study in Sect. 5.

## The institutional framework

The classic Murabaha facility is a forward sale and is related to TC.^17^ We first discuss the intricacies of the Islamic value system (in Sects. 2.1 and 2.2) and link the same to the TC literature (in Sect. 2.3).

### The Islamic perspective on plain vanilla debt financing

**Fig. 2:**
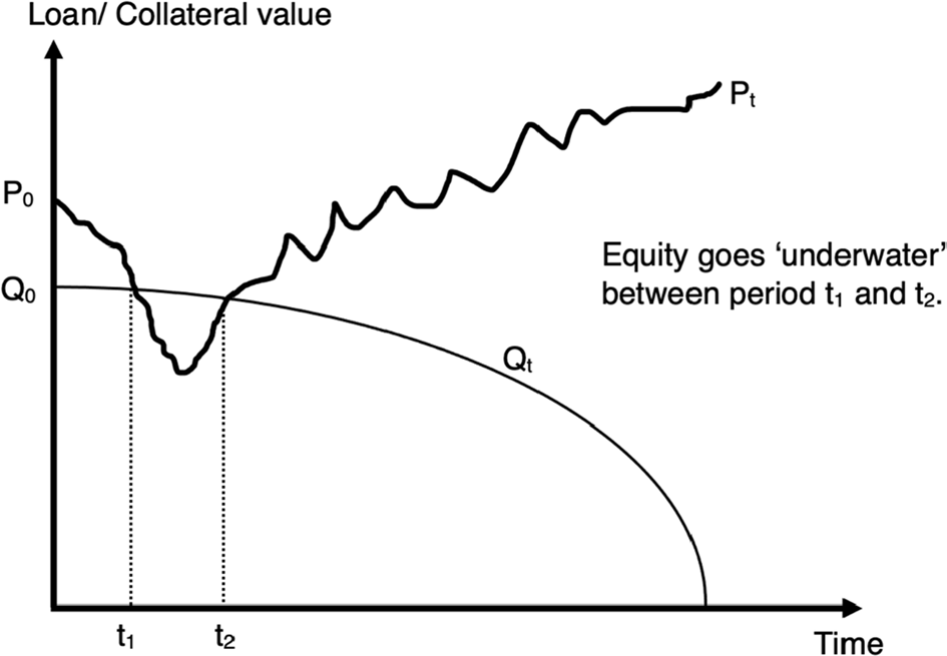
Risk-shifting. *Note*: Risk-shifting refers to the transfer of the downside risk by the borrower to the financier when the former’s equity is ‘underwater.’ That is, when the value of a company’s asset $\left({P}_{t}\right)$ is lower than its debt obligation $\left({Q}_{t}\right)$ as observed in the interval $\left({t}_{1}, { t}_{2}\right)$. This debt obligation is evaluated as follows. ${Q}_{t}={Q}_{0}\left(\frac{1-{\gamma }^{(T-t)}}{1-{\gamma }^{T}}\right)$, where ${Q}_{0}$ is the initial value of funds disbursed by the financier, $\gamma$ is the discount rate, and $T$ is the tenure of the long-term debt (or contemporary Murabaha) facility. The Excel Spreadsheet can be used to trace the $\left({Q}_{0}-{Q}_{t}\right)$ frontier with numerical values. *Source*: Wojakowski et al. (2019)

**Fig. 3:**
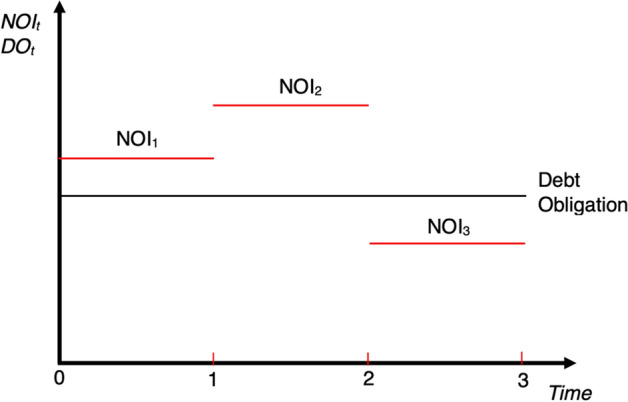
Underinvestment. *Note*: Underinvestment refers to the tendency of borrowers to reject profitable (i.e., the positive net present value—NPV) projects if the increase of wealth mainly benefits the financiers. This yields the condition where the borrower's net operating income (NOI) is lower than its Debt Obligations (DOs), as illustrated in Period 3. *Source*: Wojakowski et al. (2019)

Islamic law condemns *“trading two goods of the same kind [genus] in different quantities, where the increase is not a proper compensation”* (El-Gamal 2006, p. 49). According to this terminology, a debt that exchanges money for money is considered a prohibited transaction. Ebrahim et al. (2017) employ a rational expectations framework to rationalize the prohibition of debt to the potential harm ensuing from the: (i) expropriation of either borrower’s or lender’s wealth resulting from either risk-shifting (see Fig. 2) or underinvestment (see Fig. 3); (ii) fragility of macroeconomy; and (iii) financial exclusion of the poor.^18^

As fixed-claim debt is banned, the Islamic Finance (IF) industry is purported to develop products without embodying any increase (or excess) in receipt over the payment of the same genus (as currency). Ironically, the use of the contemporary Murabaha facility is designed to circumvent the proscription of this form of debt. This is conducted by supposedly construing a credit sale facility as a substitute for bank debt through the ‘sale’ of real (or tangible) assets (which do not share the same genus as currency) for money later. This is rationalized as permissible in accordance with the *Qur’ānic* injunction “*…God has permitted trade and forbidden debt-based transactions…*” (Q2:275).^19^

### Is Islamic banking a new paradigm?

Islamic banking was conceived by Uzair (1976) as employing the medieval profit-sharing facility. This financial structure relied on a quasi-equity-based (i.e., risk-sharing) model endowing growth and stability to develop the economy. Implementing this model was believed to be a breakthrough to stem the tide of the underdevelopment of the Muslim world. However, this model did not work in practice due to asymmetric information and transaction costs in conjunction with a poorly functioning legal infrastructure (Khan 2010). Therefore, some adjustments were needed to make IF more pragmatic and competitive than its conventional counterpart. These endeavors thus yielded the legal device of contemporary Murabaha proposed by the Islamic Development Bank (IDB) laureate Sami Hamoud in 1976 (Kahf 2013). That device modified the concept behind the medieval credit sale facility of classical Murabaha, which supposedly initiated a new paradigm in banking. The contemporary Murabaha facility is currently employed for liquidity management, Islamic bond issuance, and credit-sale transactions.^20^ Although this facility is not inconsistent with the legal-based rulings, it has unfavorable implications in the context of the objectives of Islamic law.^21^

### The Role of TC and the classic Murabaha

TC plays a vital role in short-term financing. Here we highlight various reasons why TC is deemed an alternative to bank loans.^22^

#### Financing advantage perspective

Suppliers offering credit to buyers enjoy numerous financing advantages over financial institutions (Ng et al. 1999; Nilsen 2002; Giannetti et al. 2011; Albuquerque et al. 2015; Shenoy and Williams 2017). This is especially true given a weak rule of law and firms not having legal recourse in case of defaults (Burkart and Ellingsen 2004). In addition, suppliers, as providers of TC, may operationalize these effects better than financial institutions. For example, first, suppliers may be able to decipher information about their buyers quickly and at a lower cost than financial institutions (see Smith 1987; Brennan et al. 1988; Ng et al. 1999; Chod 2017). These reasons, coupled with establishing a long-term buyer–supplier relationship, may supersede an additional operational cost incurred by financial institutions emanating from the credit risk evaluation (Biais and Gollier 1997; Giannetti et al. 2011; Kim and Shin 2012; Itzkowitz 2015). Second, suppliers may have an advantage over financial institutions in providing TC to buyers as they may threaten to stop future sales of intermediate goods, especially in cases where there are few suppliers (Cuñat 2007). Third, the TC provision confers an advantage to suppliers in salvaging the value of assets in case of buyer’s default (Fabbri and Menichini 2010; Sautner and Vladimirov 2018). This is because the supplier may reclaim the goods supplied and resell these at a lower cost, given that it already has a network for selling its products. We believe that this ability to restore the value of goods in default alleviates risk-shifting plaguing long-term debt (see again Fig. 2). Moreover, from the buyer’s point of view, TC can also be an effective channel for signaling the firm’s quality to investors (Aktas et al. 2012).

#### Firm profitability perspective

Offering TC to insure customers against liquidity risk exposes the selling firm to the risk of delayed payment or default (Petersen and Rajan 1997; Cuñat 2007). Reducing this risk requires the firm to incur administrative costs for assessing credit risk and structuring the inherent vendor financing contract. TC also locks up a large amount of capital in accounts receivable, thereby deterring the selling firms’ ability to undertake value-enhancing investment projects. This may compel the firm to obtain capital at a higher cost, thereby diminishing its profitability (Kieschnick et al. 2013; Aktas et al. 2015; Ben-Nasr 2016; Afrifa et al. 2021).

#### Price discrimination perspective

TC allows vendors to price-discriminate their buyers based on their credit history, especially when the supplier concentration is high (Chod et al. 2019). TC may be advanced even if the supplier does not have a financing advantage over financial institutions (Brennan et al. 1988; Petersen and Rajan 1997). An alternative view is that the supplier may not provide TC to a risky buyer solely because of short-term goals. Still, it may have a long-term objective in the survival of the buying firm, thereby protecting the value of its implicit equity stake (Wilner 2000). This long-term relationship in the business supply chain helps alleviate the underinvestment issue afflicting long-term debt (see again Fig. 3). This is because TC is cyclical with the economy. That is, it increases with economic expansion and decreases (but does not cease) with the subsequent contraction. This does not constrain businesses in contrast to long-term debt. The supplier may also discriminate and subsidize risky buyers with low-financing rates to take advantage of charging higher rates to the firms that survive in the future.

#### Product warranty perspective

Some industries may require TC to serve as a guarantor of product quality (Long et al. 1993; Emery and Nayar 1998). This is particularly true for differentiated input products or services (Giannetti et al. 2011; Chod et al. 2019). Accordingly, the supplier may willingly provide credit for products that need more quality assurance for their inputs, e.g., high-technology or newly developed products, to allow sufficient time for the buyer to test the product (Long et al. 1993).

#### Transaction costs perspective

TC exists to reduce the transaction costs of paying bills on delivery (Ng et al. 1999; Nilsen 2002). Depending on the nature of the products, transaction costs for some industries may be higher as they may need more frequent deliveries of inputs or have to build up extensive inventories to maintain smooth production cycles than others. Instead of paying every time goods are delivered or stored, a buyer might want to pay for them only periodically.

We conclude this subsection by emphasizing that TC is a viable alternative to the contemporary banking Murabaha-based debt facility. We link the same to the classic Murabaha facility in restructuring an Islamic financial architecture, as elaborated in Sect﻿.﻿ 3 below.

## The way forward

This section first discusses the idea behind reforming Islamic banking by reconstruing the contemporary Murabaha facility as a TC offered by a seller of a specialized intermediate good in the *real* (i.e., business) sector of the economy. Our study thus integrates the TC literature to suggest theoretical pricing of the classic Murabaha facility and, subsequently, a financial architecture.

### Reconstruing the classic Murabaha facility as a TC

Conceptualizing working capital financing with the contemporary Murabaha facility within the *financial* sector is inappropriate (Khan 2009). This is because arbitrage forces any so-called ‘Islamic’ benchmark rate to converge to the conventional rates (Azad et al. 2018). An alternative is to hypothesize the classic Murabaha facility as a TC in the *real* sector of the economy. However, this is tantamount to reinstating its original economic rationale, i.e., to increase the demand for the goods sold on credit and help complete the market (Sen 1998). To this end, we restore the classic Murabaha facility in the working capital framework. This necessitates connecting our model with that of TC or vendor financing (see Brennan et al. 1988; Shenoy and Williams 2017) and entails pricing the demand and supply in our working capital setting.

**Fig. 4:**
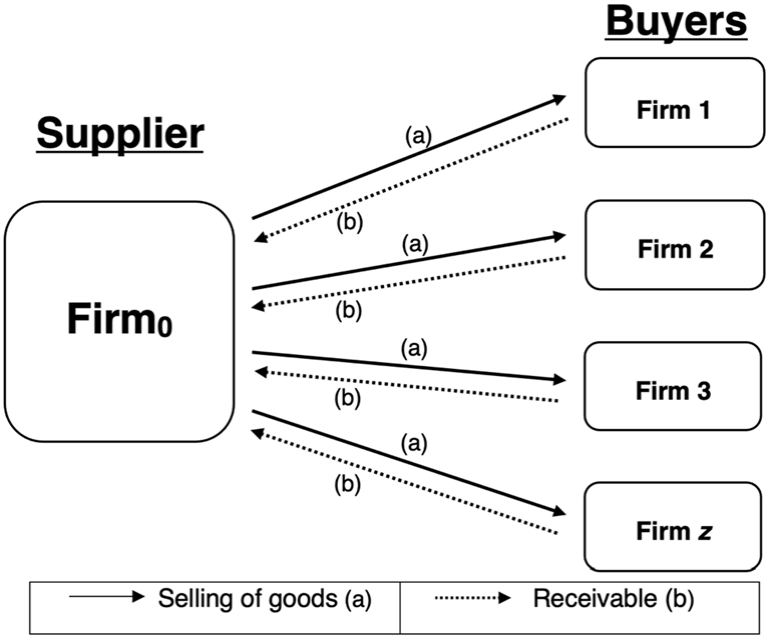
TC-Murabaha financing mechanism illustrating links between a supplier and buyers

Figure 4 illustrates the gist of our TC-based Murabaha financing model where identical firms (i.e., businesses) (${\mathrm{Firm}}_{0}$) each sell their intermediate (final) goods (or services) on credit using this risk-sharing facility to $Z$ identical buying firms (${\mathrm{Firm}}_{i}, i\in \left[1,Z\right]$) in the *real* sector of the economy and prices it in equilibrium in accordance with the supply and demand mechanism, instead of employing an interest rate benchmark. It should be noted that the selling can involve tangible assets as well as intangible services. This expands the scope of the selling firm.^23^ The selling (buying) firm will record the goods sold as accounts receivable (accounts payable) and receive (pay) the payments at some date later. It is thus equivalent to working capital financing offered by a selling firm to buying firms.

Our proposed TC-Murabaha model has several advantages over the contemporary Murabaha approach. First, from the selling firm’s perspective, TC ensuing from our classic Murabaha leads to its value optimization as it is an integral part of its working capital policy. Our proposal is consistent with Aktas et al. (2015) and Afrifa et al. (2021). They find an optimum net working capital (NWC) in US corporations at a level where firms improve their operating performance and market value. In the same vein, Ben-Nasr (2016), in a sample of 54 countries, illustrates a U-shaped relationship between NWC and the value of a firm. The channels of the relationship between NWC and firm performance can be perceived in many ways, as discussed in Sect. 2.3. In addition, the TC-Murabaha can also stabilize product demand in the recessionary period (Emery 1987) and thus enhance the long-term relationship between the selling and buying firms (Summers and Wilson 2002).

Second, from the perspective of the buying firms, TC is an alternative to short-term financing and a means of offering a warranty for product quality, also discussed in Sect. 2.3. The TC-Murabaha facility can accommodate both of these perspectives.

Finally, from a religious (and hence cultural) as well as ethical perspective, the TC-Murabaha remedies chronic issues of contemporary Murabaha financing ensuing from the: (i) ownership of the tangible goods; and (ii) fragile facility with benchmark pricing issues. This is because both of these violate the spirit of Islamic law. The former is easily tackled, as the intermediate goods/services sold originate from the selling firm’s inventory (or capacity to deliver the requisite service). The latter is solved using the pricing model, as illustrated by the supply and demand mechanism in the following subsection. This solution thus re-embeds the TC-Murabaha within the *real* sector of the economy and hence mitigates the fiduciary risk, impairing the reputation of the Islamic banking industry. Again, this is because the model complies with the form as well as the substance of the law as the financing is conducted in the *real* sector of the economy.

The TC-Murabaha facility entails higher opportunity costs of capital (Ben-Nasr 2016). Therefore, once a selling firm has decided to provide TC, it must do so at an optimum level of NWC, as suggested by Aktas et al. (2015), Ben-Nasr (2016), and Afrifa et al. (2021). Furthermore, it must also meticulously price the TC-Murabaha facility to ensure a value-enhancing price for both the selling and buying firms. The following subsection elaborates on this issue.

### Modeling the TC-Murabaha facility in the *real* sector of the economy

Here, we develop an equilibrium model to evaluate the TC-Murabaha discount (profit) rate (${m}^{*}$) incorporating the price elasticities of the product from the perspective of the selling ${\mathrm{Firm}}_{0}$ (${\epsilon }_{S}$), as well as that of the buying (${\mathrm{Firm}}_{i}$) (${\epsilon }_{B}$).^24^ We do this by extending the R&M model and integrating the selling firm (i.e., supply) side optimization function in addition to the buyers’ (i.e., demand) side to solve for the equilibrium TC rate.^25^

We assume a single-period setting where each ${\mathrm{Firm}}_{0}$ sells $Q$ units of a product to several buyers. The goal of the selling firm is to set the terms of its credit (i.e., Murabaha) sale as ${\alpha }_{S}$ and a net period of $n$. That is, ${\alpha }_{S}\%$ discount for immediate or full payment at the end of $n$ days. The selling firm faces a variable cost of $v$ per dollar of sales and sells at a price $P$ to its credit buyers at a time $n$. Production is assumed to take place instantaneously at the time of sale.

We assume further that a portion $\theta$ of buyers decide to buy the goods/services on the spot, while a portion $(1-\theta )$ buy the same on credit. Of the buyers who opt for credit, a proportion $\omega$ pay in one lump sum on time at $n$, while $(1-\omega )$ default. This $(1-\omega )$ term incorporates risk-sharing in our pricing model. We further assume that ${k}_{S}$ and ${k}_{B}$ represent the opportunity costs of capital for the selling firm and buying firms, respectively.

In developing our analysis, we first model the optimal TC rate from the selling firm’s perspective, followed by that of the buying ones. The two models are then simultaneously solved to determine the equilibrium TC rate. This is then linked to the equilibrium Murabaha rate derived in “Appendix A”. Finally, we illustrate our model solution with numerical simulations.

#### Modeling the supply-side

This subsection models the selling firm.^26^ The objective of this ${\mathrm{Firm}}_{0}$ is to maximize the present value of its future cash flow $({V}_{S}$) with respect to the firm’s TC rate (${\alpha }_{S}$)

1 $$\underset{{\alpha }_{S}}{\text{max}}\hspace{0.33em}{V}_{S}({\alpha }_{S})\hspace{0.33em},$$

subject to the following constraint

2 $${V}_{S}=\theta pQ+(1-\theta )\omega PQ(1+{k}_{S}{)}^\frac{n}{360}-vPQ,$$

where $Q$ is the total quantity supplied and

3 $$p=(1-{\alpha }_{S})P$$

is the discounted price offered to cash customers. The terms on the right-hand side (RHS) of Eq. (2) illustrate: (i) payments made by cash buyers; (ii) present value (PV) of payments made by credit buyers; and (iii) costs incurred by the selling firm.

To maximize ${V}_{S}$ with respect to ${\alpha }_{S}$ we evaluate the usual First Order Necessary Condition (FONC—hereafter)

4 $$\partial {V}_{S}({\alpha }_{S})/\partial {\alpha }_{S}=0\hspace{0.33em}.$$

Dividing the resulting expression by $P$ gives

5 $$(1-{\alpha }_{S}-\omega \beta )Q\frac{\partial \theta }{\partial {\alpha }_{S}}+(1-\theta )\beta Q\frac{\partial \omega }{\partial {\alpha }_{S}}-\theta Q+((1-{\alpha }_{S})\theta +(1-\theta )\omega \beta -v)\frac{\partial Q}{\partial {\alpha }_{S}}=0\hspace{0.33em},$$

where we used notation $\beta$ for the discount factor $(1+{k}_{S}{)}^{-\frac{n}{360}}$, with ${k}_{S}$ representing the opportunity cost of capital of the selling firm. This is a function of the partial derivatives $\frac{\partial \theta }{\partial {\alpha }_{S}}$, $\frac{\partial \omega }{\partial {\alpha }_{S}},$ and $\frac{\partial Q}{\partial {\alpha }_{S}}$. We rationalize the three derivatives as follows.

R&M classify the above first two derivatives as relating to the behavior of the buyers. The first one specifies the proportion ($\theta$) of cash buyers in our setting. It is argued that as the discount rate ($\alpha$) increases, buyers avoid buying on credit. That is, they seek alternative sources of financing and opt to pay cash for their purchases. This implies that^27^

6 $$\frac{\partial \theta }{\partial \alpha }>0\hspace{0.33em}.$$

To derive the optimum TC rate (${\alpha }^{*}$) the relationship between $\alpha$ and $\theta$ needs to be further simplified. It must be such that when $\alpha =0$, then $\theta =0,$ and when $\alpha$ is very high, then $\theta =1$. This implies that $\theta$ is an increasing function of $\alpha$ and is between the two extremes. A simple relationship capturing this is a linear one given as follows

7 $$\theta =\gamma \alpha \hspace{0.33em},$$

where $\gamma >0$ is a positive constant. Furthermore, since $\alpha ={\gamma }^{-1}$ implies that 100% of buyers switch to cash purchases ($\theta =1$). The following range must be observed for meaningful $\alpha^{\prime }$*s*

8 $$0<\alpha <\overline{\alpha }={\text{min}}\left\{1,{\gamma }^{-1}\right\}\hspace{0.33em}.$$

The second derivative refers to the proportion ($\omega )$ of credit buyers who do not default. Unlike that of $\theta$, the relationship of $\omega$ with respect to change in $\alpha$ is not straightforward. Two opposing effects are feasible. They are described as follows. First, consistent with the impact on $\theta$, an increase in $\alpha$ encourages buyers to purchase with cash instead of using the TC facility. This lowers the number of credit buyers who pay on time. Second, the higher the number of buyers making cash payments reduces the number of those who default. We argue that these two offsetting effects cancel each other, resulting in a coefficient $\omega$ which remains stable through the process of changing $\alpha$. This yields

9 $$\frac{\partial \omega }{\partial \alpha }=0\hspace{0.33em}.$$

Finally, a specification of $\frac{\partial Q}{\partial \alpha }$ must be incorporated into our analysis. The supply side is conceptualized by linking the quantity supplied ($Q$) to the elasticity of supply ${\epsilon }_{S}$. From the perspective of the supplier, a higher discount rate ${\alpha }_{S}$ has an enhanced negative impact on cash sales $S=pQ$. That is, a higher discount rate ${\alpha }_{S}$ results in a lower unit price $p=(1-{\alpha }_{S})P$. Furthermore, a lower unit price ($p$) results in a smaller quantity ($Q$) supplied by the selling firm. Introducing the elasticity of supply

10 $${\epsilon }_{S}=\frac{\partial Q}{\partial p}\frac{p}{Q}>0\hspace{0.33em},$$

we can write

11 $$\frac{\partial Q}{\partial p}>0\Rightarrow \frac{\partial S}{\partial {\alpha }_{S}}=-PQ-Pp\frac{\partial Q}{\partial p}=-PQ(1+{\epsilon }_{S})<0\hspace{0.33em}.$$

Since elasticities of supply are generally positive ${\epsilon }_{S}>0$, a decrease in price $\partial p<0$ coupled with lowered quantity offered ($\partial Q<0$, on the supply-side) yields a lower sales figure. Equivalently, an increase in offered discount ($\partial {\alpha }_{S}>0$) yields a decrease in the quantity offered ($\partial Q<0$) by the supplier, negatively impacting sales $S$.^28^

Furthermore, we have

12 $$\frac{\partial Q}{\partial {\alpha }_{S}}=\frac{\partial Q}{\partial p}\frac{\partial p}{\partial {\alpha }_{S}}=-\frac{\partial Q}{\partial p}\frac{p}{Q}\frac{Q}{p}P=-{\epsilon }_{S}\frac{Q}{1-{\alpha }_{S}}\hspace{0.33em}.$$

Substituting Eqs. (7), (9), and (12) back into Eq. (5) and dividing by $Q$ we obtain

13 $$(1-{\alpha }_{S}-\omega \beta )\gamma -\gamma {\alpha }_{S}-((1-{\alpha }_{S})\gamma {\alpha }_{S}+(1-\gamma {\alpha }_{S})\omega \beta -v)\frac{{\epsilon }_{S}}{1-{\alpha }_{S}}=0\hspace{0.33em}.$$

Multiplying both sides by $1-{\alpha }_{S}$ gives a quadratic equation in ${\alpha }_{S}$

14 $${a}_{S}{\alpha }_{S}^{2}+{b}_{S}{\alpha }_{S}+{c}_{S}=0\hspace{0.33em},$$

where

15 $${a}_{S}= \gamma \left({\epsilon }_{S}+2\right) ,$$

16 $${b}_{S}=-\gamma \left({\epsilon }_{S}+3-{\beta }_{S,n}\omega \left({\epsilon }_{S}+1\right)\right) ,$$

17 $${c}_{S}=\gamma +v{\epsilon }_{S}-{\beta }_{S,n}\omega \left({\epsilon }_{S}+\gamma \right) ,$$

and ${\beta }_{S,n}=(1+{k}_{S}{)}^{-n/360}.$ The root of the above quadratic Eq. (14), which falls within the valid range, as in Eq. (8), i.e., $(0,{\overline{\alpha }}_{S})$, is

18 $${\alpha }_{S}^{*}=\left(-{b}_{S}-\sqrt{{b}_{S}^{2}-4{a}_{S}{c}_{S}}\right)/2{a}_{S}\hspace{0.33em}.$$

#### Modeling the demand-side

The objective of the buying firms is also to maximize the present value of their future cash flow with respect to the discount rate, ${\alpha }_{B}$. The aggregate present value of future cash flows for all buyers is given as follows

19 $$\underset{{\alpha }_{B}}{\text{max}}\hspace{0.33em}{V}_{B}({\alpha }_{B})\hspace{0.33em},$$

subject to the following constraint

20 $${V}_{B}({\alpha }_{B})=\tau PQ-\theta pQ-(1-\theta )\omega PQ{\beta }_{B,n}\hspace{0.33em},$$

where ${\alpha }_{B}$ is a discount rate offered to buyers,

21 $$p=p({\alpha }_{B})=P(1-{\alpha }_{B})$$

is the discounted price, and

22 $${\beta }_{B,n}=(1+{k}_{B}{)}^{-\frac{n}{360}}$$

is the buyers’ discount factor, where ${k}_{B}$ is the buyers’ opportunity cost of capital. The first term of Eq. (20) represents the perceived worth of the goods (or services) purchased by the buyers. The value of $\tau$ is positive and equals one (i.e., unity) if the goods are valued at par. Their worth may also be less or more than one if they are valued at a discount or premium, respectively. The second term represents the spot price paid (net of discount) by the cash buyers. Finally, the last term reflects the discounted cost paid by credit buyers.

The necessary steps to derive the optimal buyers’ discount rate follow those employed for deriving the optimal supplier’s discount rate. First, to maximize ${V}_{B}$ with respect^29^ to ${\alpha }_{B}$ we compute the demand-side FONC

23 $$\partial {V}_{B}({\alpha }_{B})/\partial ({\alpha }_{B})=0\hspace{0.33em}.$$

Dividing the resulting expression by $P$ yields

24 $$(\tau -\theta \left(1-{\alpha }_{B}\right)-\left(1-\theta \right)\omega {\beta }_{B,n})\frac{\partial Q}{\partial {\alpha }_{B}}+\left(\omega {\beta }_{B,n}+{\alpha }_{B}-1\right)Q\frac{\partial \theta }{\partial {\alpha }_{B}}-(1-\theta ){\beta }_{B,n}Q\frac{\partial \omega }{\partial {\alpha }_{B}}+\theta Q=0.$$

The buyers’ side reflects the same assumptions as those of the supplier in terms of $\theta ({\alpha }_{B})$ and $\omega$ (see again Eqs. (7) and (9)). Furthermore, consistent with the supply-side analysis, the total quantity demanded ($Q$) is assumed to be a function of the effective spot price $p$ (i.e., discounted price $P$). The elasticity of demand is given below

25 $${\epsilon }_{B}=\frac{\partial Q}{\partial p}\frac{p}{Q}\le 0\hspace{0.33em}.$$

The impact of a higher discount rate ${\alpha }_{B}$ on the spot price of the product is contingent on its price elasticity (${\epsilon }_{B})$. We can express this as a reaction to an increase in ${\alpha }_{B}$ on sales $S$ as given below

26 $$\frac{\partial S}{\partial {\alpha }_{B}}>0\iff -PQ-Pp\frac{\partial Q}{\partial p}>0\iff \frac{\partial Q}{\partial p}<0\iff -\frac{Q}{p}<0\hspace{0.33em}.$$

That is, sales increase only if the quantity demanded increases sufficiently enough $\partial Q>0$ following a decrease in price $\partial p<0$. Incorporating the price elasticity of demand translates into

27 $$\frac{\partial S}{\partial {\alpha }_{B}}>0\iff {\epsilon }_{B}<-1\hspace{0.33em}.$$

The demand for an elastic product $({\epsilon }_{B}<-1)$ responds to the changes in ${\alpha }_{B}$ more extensively. That is, an increase in ${\alpha }_{B}$ leads to an increase in sales $S$. On the other hand, the demand for an inelastic product $({\epsilon }_{B}>-1)$ is less sensitive to a change in price. That is, an increase in ${\alpha }_{B}$ leads to a decrease in sales $S$

28 $$\frac{\partial S}{\partial {\alpha }_{B}}<0\iff {\epsilon }_{B}>-1\hspace{0.33em}.$$

Finally, a unitary elastic good (or service) does not affect the level of sales $S$

29 $$\frac{\partial S}{\partial {\alpha }_{B}}=0\iff {\epsilon }_{B}=-1\hspace{0.33em}.$$

After incorporating the earlier behavioral assumptions, as in Eqs. (7) and (9), and the elasticity of demand (${\epsilon }_{B}$) into Eq. (24), we multiply both sides of the resulting equation by $-(1-{\alpha }_{B})/Q$. This yields

30 $$\tau {\epsilon }_{B}-\gamma ({\alpha }_{B}(2+{\epsilon }_{B})-1)(1-{\alpha }_{B})-{\beta }_{B,n}\omega (\gamma (1-{\alpha }_{B})+{\epsilon }_{B}(1-\gamma {\alpha }_{B}))=0\hspace{0.33em}.$$

As before, to solve for ${(\alpha }_{B}$) we simplify this as a quadratic equation in ${\alpha }_{B}$

31 $${a}_{B}{\alpha }_{B}^{2}+{b}_{B}{\alpha }_{B}+{c}_{B}=0\hspace{0.33em},$$

where

32 $${a}_{B}=\gamma \left({\epsilon }_{B}+2\right) ,$$

33 $${b}_{B}=-\gamma \left({\epsilon }_{B}+3-{\beta }_{B,n}\omega \left({\epsilon }_{B}+1\right)\right),$$

34 $${c}_{B}= \gamma +\tau {\epsilon }_{B}-{\beta }_{B,n}\omega \left({\epsilon }_{B}+\gamma \right) ,$$

and ${\beta }_{B,n}=(1+{k}_{B}{)}^{-n/360}$. We then select the root of the above quadratic Eq. (31), which falls within the valid range (8), i.e., ($0,\overline{{\alpha }_{B}}$). That is

35 $$\left(-{b}_{B}-\sqrt{{b}_{B}^{2}-4{a}_{B}{c}_{B}}\right)/2{a}_{B}\hspace{0.33em}.$$

Compared to the quadratic Eq. (14) for suppliers, the quadratic Eq. (31) for buyers incorporates the demand-side price elasticity ${\epsilon }_{B}<0$, the buyers’ opportunity cost of capital ${k}_{B}$ (via the buyers’ discount factor ${\beta }_{B,n}$), as well as the buyers’ perceived worth of goods $\tau$. The coefficients $\left\{{a}_{B},{b}_{B},{c}_{B}\right\}$, structurally mirror those for the suppliers, $\left\{{a}_{S},{b}_{S},{c}_{S}\right\}$. However, there is a change in sign in the elasticities reflecting the demand-side.

#### Market clearing condition

For markets to clear, the bid (${\alpha }_{B}$) and the ask (${\alpha }_{S}$) prices of TC should equal, along with the respective quantities bought on credit. This yields:

36 $${\alpha }_{S}={\alpha }_{B}=\alpha \hspace{0.33em}.$$

Since prices of financial facilities and discount rates are inverse of each other, the above equation implies that the bid price equals the highest buy order, while the ask price equals the lowest sell order. The above requirement can be combined with conditions as in Eqs. (14) and (31) for the supplier’s and buyers’ sides, respectively. In general, when ${k}_{S}\ne {k}_{B},$ ${\epsilon }_{B}<0$, and ${\epsilon }_{S}>0$, these two quadratic equations can be integrated to isolate the common market-clearing TC rate ($\alpha$). We accomplish this by subtracting Eq. (14) from Eq. (31) to get

37 $$a{\alpha }^{2}+b\alpha +c=0\hspace{0.33em},$$

where the quadratic equation’s coefficients are simplified as follows

38 $$a={a}_{B}-{a}_{S}=\gamma \left({\epsilon }_{B}-{\epsilon }_{S}\right)\hspace{0.33em},$$

39 $$b={b}_{B}-{b}_{S}=-\gamma \left({\epsilon }_{B}-{\epsilon }_{S}-{\beta }_{B,n}\omega \left({\epsilon }_{B}+1\right)+{\beta }_{S,n}\omega \left({\epsilon }_{S}+1\right)\right)\hspace{0.33em},$$

40 $$c={c}_{B}-{c}_{S}=\tau {\epsilon }_{B}-v{\epsilon }_{S}-{\beta }_{B,n}\omega \left({\epsilon }_{B}+\gamma \right)+{\beta }_{S,n}\omega \left({\epsilon }_{S}+\gamma \right)\hspace{0.33em}.$$

This quadratic equation provides an optimal equilibrium value of the TC-Murabaha rate involving the product price elasticity of the selling firm and the buying ones. Thus, the optimal ${\alpha }^{*}$ can be obtained in the range ($0,\overline{\alpha }$) by solving Eq. (37), as given below^30^

41 $${\alpha }^{*}=\left(-b-\sqrt{{b}^{2}-4ac}\right)/2a\hspace{0.33em}.$$

The above rate is used to evaluate the optimal TC-Murabaha discount rate (derived in “Appendix A”) as follows

42 $${m}^{*}=\frac{{\alpha }^{*}}{1-{\alpha }^{*}}\hspace{0.33em}.$$

Thus, our result given by Eq. (42) is in the spirit of Lam and Chen (1986, p. 1146), who state that “*For a value-maximizing firm, the optimality condition for pricing requires equating the marginal value of accounts receivable to the marginal value of the cost of production…*”.

## Results and discussion

### Key result

#### Proposition

*The TC-Murabaha discount rate is a function of not only the prevailing opportunity costs of capital of both the selling firm and the buying firms but also incorporates the price elasticities of the product. This has the potential to offer a ‘Murabaha’ rate even lower than the opportunity costs of capital of the financiers and buyers, especially in periods of monetary contractions. Moreover, since the elasticities of buyers are heterogeneous in the real world, this allows the selling firm to price-discriminate among their buyers. This feature enables the TC-Murabaha to curtail the deadweight loss stemming from market inefficiencies, thus inducing welfare gains for society.*

The above result is derived from a competitive market framework. Nonetheless, we are of the view that our general conclusion may not deviate from other market structures as they are obtained under the economies of scale and scope of businesses (i.e., trading or commercial organizations) operating in the *real* sector of the economy.^31^ Our result also exemplifies the deeper meaning of *trade* or *commerce* and sets it apart from subterfuges employed by the Islamic banking industry in the *financial* sector of the economy. This result has ramifications beyond the working capital environment of businesses.^32^ This is illustrated with numerical simulations in Sect. 4.2 below.

Our result is consistent with the economic intuition of credit sales in helping complete the market as espoused in Sen (1998). However, our findings contradict the contemporary Murabaha rate derived from the simple textbook formula of the market interest rate plus a risk premium (in conjunction with a term premium).^33^ The reason for our improved result is attributed to the optimization of the welfare of both competing businesses (i.e., buyer and seller), leading to the economies of scale and scope in the *real* sector of the economy. This contrasts with the contemporary IB, which operates in the highly efficient *financial* sector where arbitrage forces it to offer market-driven rates.^34^

Furthermore, the above innovative proposition responds to the call for advancing IB by re-embedding the economic rationale and, thus, the objective of Islamic law. Our results have significant ramifications, especially in the current coronavirus (and energy) crisis, as they do not burden businesses with long-term debt and yield a very efficient and resilient financial architecture, as elaborated in Sect. 4.3.^35^

Since the framework suggested by us does not currently exist, we cannot test the prognosis of our model empirically. We, therefore, resort to numerical simulation in the following subsections to illustrate the central prediction of our study. That is, the efficiency of the proposed IB architecture constructed by replacing the controversial banking Murabaha facility with TC.

### Proof of proposition employing numerical simulation

#### The effect of price elasticity on the optimum TC-Murabaha Discount Rate

**Fig. 5:**
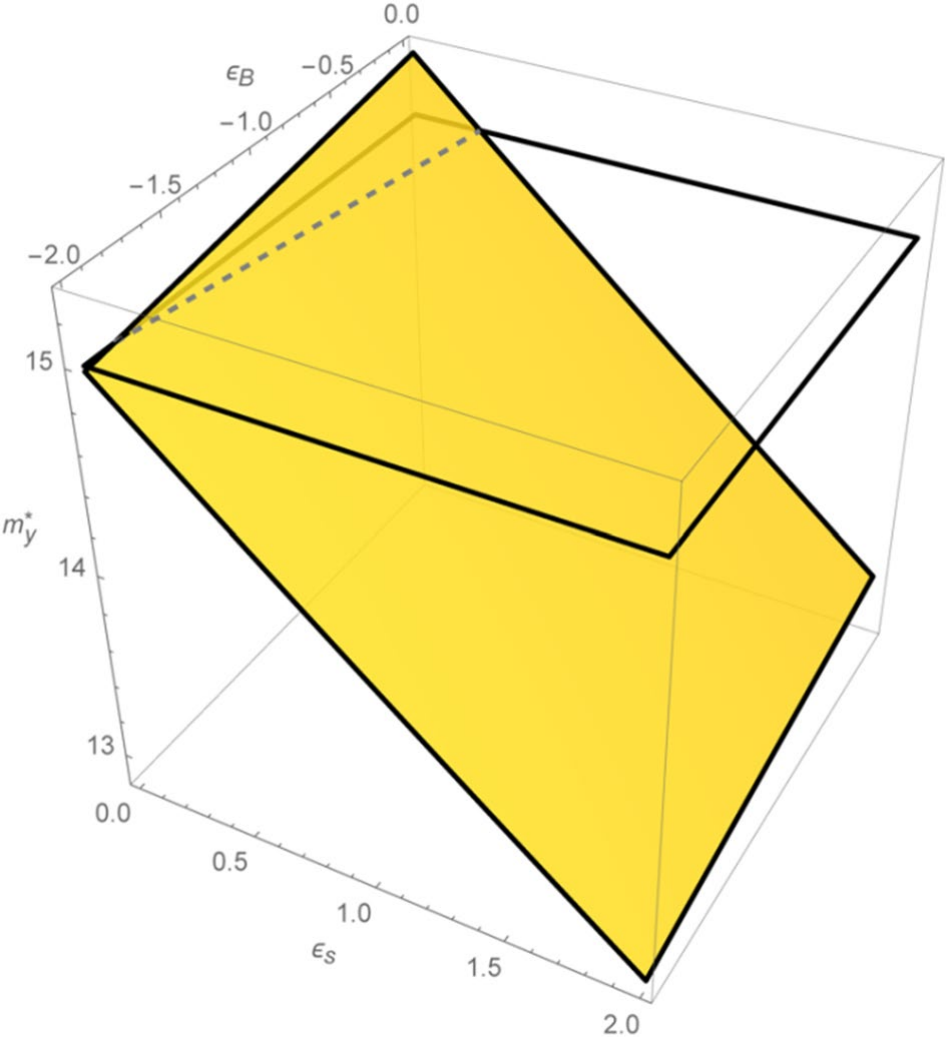
Optimum TC-Murabaha rates across various price elasticities. *Note*: The variables ${m}_{y}^{*}, {\epsilon }_{B},$ and ${\epsilon }_{S}$ represent the optimum annualized TC-Murabaha mark-up rate and the respective elasticities of demand and supply. The above figure illustrates: (a) the TC-Murabaha rate (${m}_{y}^{*}$) can be lower than the opportunity cost of capital (represented as a horizontal translucent surface at the $k=15\%$ level) for most of the price elasticity levels ${(\epsilon }_{B}, {\epsilon }_{S})$; (b) a negative relationship between the levels of price elasticity ${(\epsilon }_{B}, {\epsilon }_{S})$ and optimum TC-Murabaha discount rate ${m}_{y}^{*}$; (c) the minimum and maximum TC-Murabaha rates of ${m}_{y}^{*}=12.87\%$ and ${m}_{y}^{*}=15.31\%$ respectively, are derived using the highest and lowest price elasticities for the selling and buying firms (i.e., ${\epsilon }_{B}=2, {\epsilon }_{S}=-2$ and ${\epsilon }_{B}= {\epsilon }_{S}=0$, respectively)

Our model’s numerical simulation assumes the following real-world exogenous parameters: $\omega =97\%$; ${k}_{S}={k}_{B}=15\%$; $v=75\%$; $\tau =1$; $n=90$ days; and $\gamma =30$.^36^ Table 1 and Fig. 5 summarize the respective annualized discount rates ${\alpha }_{y}^{*}$’s and ${m}_{y}^{*}$’s^37^ obtained from Eqs. (41) and (42) for demand and supply price elasticities in the range ${\epsilon }_{B}\in \left[0,-2\right]$ and ${\epsilon }_{S}\in \left[\mathrm{2,0}\right]$, respectively. We compute a grid of optimal values of the discount coefficient for elasticities spaced at equally distant nodes, i.e., $\Delta {\epsilon }_{B}=\Delta {\epsilon }_{S}=0.5$. Our results illustrate the generally negative relationship between the levels of price elasticity and the optimum TC-Murabaha discount rate. This effect is most prominent in the case of the price elasticity of supply. The lowest TC-Murabaha rate is confirmed when the price elasticity of supply and demand is high ($2$ and $-2$, respectively). In this case, the optimum annualized TC rate is $11.41\%$, which is equivalent to $12.87\%$ of the optimum annualized TC-Murabaha mark-up rate, ${m}_{y}^{*}$. In contrast, the highest rate occurs when the product price elasticity of both supply and demand equals zero (i.e., inelastic case). In this situation, the equilibrium TC rate is $13.28\%$, equivalent to $15.31\%$ of the TC-Murabaha mark-up rate.

**Table 1:** Optimum annual TC-Murabaha rates under various elasticities

${\epsilon }_{S}$	${\epsilon}_{B}$									
	0		− 0.5		− 1		− 1.5		− 2	
	${\alpha }_{y}^{*}$	${m}_{y}^{*}$	${\alpha }_{y}^{*}$	${m}_{y}^{*}$	${\alpha }_{y}^{*}$	${m}_{y}^{*}$	${\alpha }_{y}^{*}$	${m}_{y}^{*}$	${\alpha }_{y}^{*}$	${m}_{y}^{*}$
2	11.65	13.19	11.59	13.11	11.53	13.03	11.47	12.95	11.41	12.87
1.5	12.06	13.71	11.59	13.63	11.93	13.55	11.87	13.47	11.81	13.39
1	12.46	14.23	12.40	14.15	12.34	14.07	12.27	13.99	12.21	13.91
0.5	12.87	14.77	12.80	14.68	12.74	14.60	12.68	14.52	12.62	14.44
0	13.28	**15.31**	13.21	**15.22**	13.15	**15.14**	13.09	**15.06**	13.02	14.97

The variable ${\epsilon }_{B}$ expresses price elasticity of demand while ${\epsilon }_{S}$ represents the price elasticity of the supply. Furthermore, ${\alpha }_{y}^{*}$ is annualized optimum TC rate, i.e., ${\alpha }_{y}^{*}=[(1+{\alpha }^{*}{)}^\frac{360}{n}-1]$, while ${m}_{y}^{*}$ represents annualized optimum TC-Murabaha rate, where ${m}_{y}^{*}={\alpha }_{y}^{*}/(1-{\alpha }_{y}^{*})$. All rates ${(\alpha }_{y}^{*}, {m}_{y}^{*})$ are expressed in percentages per annum. The bold numbers indicate that the value of the optimum TC-Murabaha rate $({m}_{y}^{*})$ is higher than the opportunity cost (*k*). The exogenous parameters assumed here are: $\omega =97\%; {k}_{S}={k}_{B}=15\%; v=75\%; \tau =1; n=90$ days; and $\gamma =30$

The above outcome exemplifies the price discrimination aspect of TC contingent on both the sellers’ and the buyers’ price elasticities. Practically, this is accomplished by a seller by offering varying TC terms to the diversity of buyers (see also Klapper et al. 2012). These results also confirm our proposition that the TC-Murabaha rate can be even lower than the opportunity cost of capital. This is because in our simulation, almost all annualized optimum TC rates (${\alpha }_{y}^{*}$) and mark-up rates (${m}_{y}^{*}$) as computed for different elasticities, are lower than $k=15\%$. All ${m}_{y}^{*}$ are lower than $k$ except when ${\epsilon }_{S}$ equals to zero.^38^ It is worth noting that, in the case of contemporary Murabaha, the opportunity cost of capital (${k}_{S}$) from the perspective of the selling firm, is equal to the minimum rate charged by banks to their debtors. In practice, however, IBs charge even a higher rate than this opportunity cost, i.e., opportunity cost plus a risk (i.e., a ‘piety’) premium.

#### Comparative static analysis: the effect of variation in the opportunity costs on the optimal TC-Murabaha rate

**Fig. 6:**
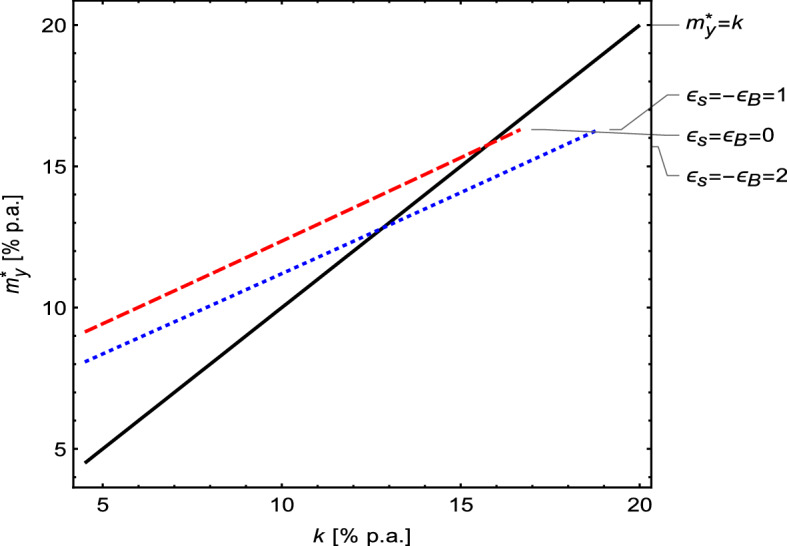
The impact of opportunity costs of capital ($k$) on TC-Murabaha rates ${m}_{y}^{*}$ for various price elasticities. *Note*: The variables ${m}_{y}^{*}, {k, \epsilon }_{B},$ and ${\epsilon }_{S}$ represent the optimum annualized TC-Murabaha mark-up rate, the opportunity cost of capital, and the respective elasticities of demand and supply. The above figure illustrates that the more elastic the product (from the perspective of both selling firms and buying ones), the higher the possibility of obtaining a TC-Murabaha discount rate lower than the opportunity cost of capital (i.e., ${m}_{y}^{*}<k$)

Figure 6 illustrates the impact of different levels of the opportunity cost of capital ($k$’s) on ${m}_{y}^{*}$.^39^ The figure suggests that the relationship between $k$’s and ${m}_{y}^{*}$’s is an increasing quasilinear function. An increase in $k$ leads to a rise in ${m}_{y}^{*}$ due to the time value of money. The price elasticities (${\epsilon }_{S}$ and ${\epsilon }_{B}$) have almost no effect on the slope of the curve. However, different values of $\epsilon$ shift the curve downward for the more elastic product and upward for the less elastic one.

Furthermore, Table 2 depicts cases (highlighted in bold) where ${m}_{y}^{*}$ s’ are lower than $k$. These occur when (i) $k$ and/or (ii) $\epsilon$ increase. In the base case, when $k$ equals 11%, ${m}_{y}^{*}$ is always higher than $k$ for all values of ${\epsilon }_{S}$ and ${\epsilon }_{B}$, except when ${\epsilon }_{S}$ equals to 2. However, when $k$ increases to $12\%$, then ${m}_{y}^{*}$ for the lower elasticity of supply of the product, i.e., ${\epsilon }_{S}=1.5$, also becomes lower than $k$ for all levels of elasticity of demand, ${\epsilon }_{B}$. Finally, when $k$ increases to $16\%$, then ${m}_{y}^{*}$ becomes lower than $k$ for all combinations of elasticity levels. Thus, the higher the value of the discount rate $k$, the lower the feasibility of the TC-Murabaha pricing rate, i.e., ${m}_{y}^{*}<k$. This shows that ${m}_{y}^{*}$ is more stable than $k$, especially in the case where $k$ increases rapidly, such as in an inflationary environment.

**Table 2:** Cases where the optimum annual TC-Murabaha rates are lower than the opportunity costs of capital

	$k=11$					$k=12$				
${\epsilon }_{S}$	${\epsilon }_{B}$					${\epsilon }_{B}$				
	0	− 0.5	− 1	− 1.5	− 2	0	− 0.5	− 1	− 1.5	− 2
2	**10.93**	**10.86**	**10.79**	**10.72**	**10.64**	**11.50**	**11.42**	**11.35**	**11.27**	**11.20**
1.5	11.42	11.35	11.28	11.20	11.13	**11.99**	**11.92**	**11.84**	**11.77**	**11.69**
1	11.92	11.85	11.77	11.70	11.62	12.50	12.42	12.35	12.27	12.19
0.5	12.43	12.35	12.28	12.20	12.13	13.01	12.93	12.86	12.78	12.70
0	12.94	12.86	12.79	12.71	12.64	13.53	13.45	13.37	13.30	13.22

	$k=13$					$k=14$				
${\epsilon }_{S}$	${\epsilon }_{B}$					${\epsilon }_{B}$				
	0	− 0.5	− 1	− 1.5	− 2	0	− 0.5	− 1	− 1.5	− 2
2	**12.06**	**11.99**	**11.91**	**11.83**	**11.76**	**12.63**	**12.55**	**12.47**	**12.39**	**12.31**
1.5	**12.57**	**12.49**	**12.41**	**12.33**	**12.26**	**13.14**	**13.06**	**12.98**	**12.90**	**12.82**
1	13.08	13.00	**12.92**	**12.84**	**12.76**	**13.65**	**13.57**	**13.49**	**13.42**	**13.34**
0.5	13.59	13.52	13.44	13.36	13.28	14.18	14.10	14.02	**13.94**	**13.86**
0	14.12	14.04	13.96	13.88	13.80	14.71	14.63	14.55	14.47	14.39

${\epsilon }_{S}$	$k=15$					$k=16$				
	${\epsilon }_{B}$					${\epsilon }_{B}$				
	0	− 0.5	− 1	− 1.5	− 2	0	− 0.5	− 1	− 1.5	− 2
2	**13.19**	**13.11**	**13.03**	**12.95**	**12.87**	**13.76**	**13.68**	**13.60**	**13.52**	**13.43**
1.5	**13.71**	**13.63**	**13.55**	**13.47**	**13.39**	**14.28**	**14.20**	**14.12**	**14.04**	**13.96**
1	**14.23**	**14.15**	**14.07**	**13.99**	**13.91**	**14.81**	**14.73**	**14.65**	**14.57**	**14.48**
0.5	**14.77**	**14.68**	**14.60**	**14.52**	**14.44**	**15.25**	**15.27**	**15.19**	**15.10**	**15.02**
0	15.31	15.22	15.14	15.06	**14.97**	**15.90**	**15.82**	**15.73**	**15.65**	**15.56**

This table assumes ${k}_{S}={k}_{B}=k$. The numbers highlighted in bold are solutions yielding ${m}_{y}^{*}<k$. All rates $({m}_{y}^{*})$ are expressed in percentages per annum. The exogenous parameters assumed here are $\omega =97\%; v=75\%; \tau =1; n=90$ days; and $\gamma =30$

The latter condition, for instance, might be observed by considering the case of ${\alpha }_{y}^{*}$ for $k=14\%$. In this case, ${m}_{y}^{*}$ for inelastic supply, i.e., ${\epsilon }_{S}=0$, is still higher than $k$. However, for unitary or elastic supply, i.e., ${\epsilon }_{S}\ge 1$, ${m}_{y}^{*}$ becomes lower than $k$. In addition, for less elastic supply, i.e., ${\epsilon }_{S}=1/2$, ${m}_{y}^{*}$ is higher than $k$, particularly when the demand is inelastic, ${\epsilon }_{B}=0$. When the demand gets more elastic, i.e., ${\epsilon }_{B}<0$, then ${m}_{y}^{*}$ becomes lower than $k$. Thus, the more elastic the product (from the perspective of both supply and demand), the higher the possibility of ${m}_{y}^{*}<k$. We conclude that the above supports our proposition that there is potential to offer TC-Murabaha rates below the opportunity costs of capital, i.e., when $k$ and/ or $\epsilon$ are high.

#### TC-Murabaha model and the business cycle

**Fig. 7:**
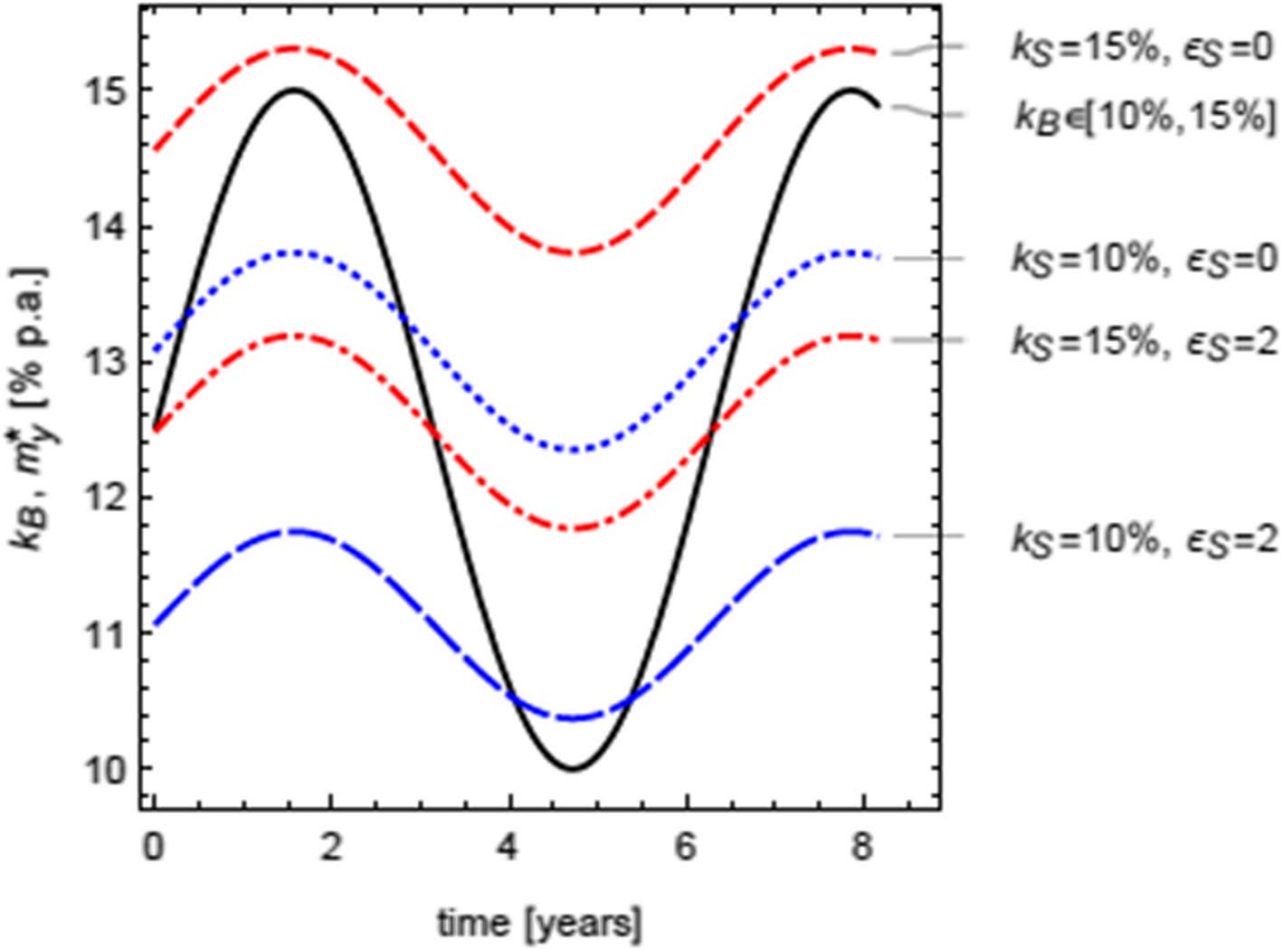
The impact of the phases of the business cycle, represented as a periodically fluctuating opportunity cost of capital of the buying firm on the annualized optimum TC-Murabaha rates. *Note*: Economic expansions are represented as a *decreasing* cost of capital to the buyer, ${k}_{B}$ (within the range [10%, 15%], black solid curve). Four possible permutations of the two values of the opportunity costs of capital of the selling firm, ${k}_{S}\in \{10\%, 15\%\}$, and the two values of the elasticity of supply, ${\epsilon }_{S}\in \{0, 2\}$, are represented (dashed red and blue curves). The four annualized optimum TC-Murabaha rates are computed as ${m}_{y}^{*}={{\alpha }_{y}^{*}/(1-\alpha }_{y}^{*})$ where ${\alpha }_{y}^{*}$ is the annualized optimum TC rate, i.e., $1+{\alpha }_{y}^{*}={(1+{\alpha }^{*})}^\frac{360}{n}$. All rates are expressed in percentages per annum. The exogenous parameters are assumed to be: $\gamma =30;v=75\%; \tau =1;n=90$ days, and the price elasticity of demand parameter is set to zero, ${\epsilon }_{B}=0$

Figure 7 illustrates the TC-Murabaha rates across varying phases of the business cycle. It shows that the opportunity cost of capital of the buying firm (${k}_{B}$) cyclically fluctuating within the range [10%, 15%] above and below the equilibrium (average) value of 12.5%. To start with, when ${k}_{B}$ increases, the economy contracts. After some time, when ${k}_{B}$ starts to decline, the economy begins to expand for buyers.^40^ Clearly, the annualized optimum TC-Murabaha rates (${m}_{y}^{*}$) closely follow the economic cycle. They are the highest and above the buyer’s cost (${k}_{B}$) when supplier’s costs are high and elasticity is low: ${k}_{S}$ = 15%, ${\epsilon }_{S}$ = 0. Conversely, when their costs are low and elasticity is high, ${k}_{S}$ = 10%, ${\epsilon }_{S}$ = 2, suppliers can offer competitive TC-Murabaha rates which are lower than the buyer’s cost of capital, i.e., ${m}_{y}^{*}<{k}_{B}$, except at the very low ebb of the ${k}_{B}$ cycle, when the economy is in full swing. As depicted, this mainly benefits the buyers as they suffer an economic squeeze due to high ${k}_{B}$. As expected, the TC-Murabaha rates (${m}_{y}^{*}$) become less attractive to buyers when their own opportunity costs of capital ${(k}_{B}$) reach the troughs, which is characteristic of the expansion phase of the economy.

#### Robustness check: relaxing the assumption of ${\varvec{k}}$

Our proposition still holds even when we relax the assumption of $k={k}_{S}={k}_{B}$. Table 3 shows ${m}_{y}^{*}$ for different values of ${k}_{S}$ and ${k}_{B}$.^41^ That is, (i) when ${k}_{S}=11\%$ while ${k}_{B}$ is varied between $11\%$ and $15\%$, and (ii) when ${k}_{B}$ = $11\%$, while varying ${k}_{S}$. The table points out a congruent result where the condition ${m}_{y}^{*}<{k}_{S}$ and ${m}_{y}^{*}<{k}_{B}$ holds in all the various price elasticities when ${k}_{S}$ or ${k}_{B}$ increase above $11\%$.

**Table 3:** Optimal annual TC-Murabaha $({m}_{y}^{*})$ rates under various opportunity costs of capital

${\epsilon }_{S}$	${\epsilon }_{B}$	${k}_{B}({k}_{S}=11\% p.a.)$					${k}_{S}({k}_{B}=11\% p.a.)$				
		11	12	13	14	15	11	12	13	14	15
0	0	12.94	13.23	13.53	**13.82**	**14.11**	12.94	13.23	13.53	**13.82**	**14.11**
0.5	0	12.43	12.72	13.01	**13.30**	**13.59**	12.43	12.72	13.01	**13.30**	**13.59**
1	0	11.92	12.21	**12.50**	**12.78**	**13.07**	11.92	12.21	**12.50**	**12.78**	**13.07**
1.5	0	11.42	**11.71**	**11.99**	**12.28**	**12.56**	11.42	**11.71**	**11.99**	**12.28**	**12.56**
2	0	**10.93**	**11.22**	**11.50**	**11.78**	**12.06**	**10.93**	**11.22**	**11.50**	**11.78**	**12.06**
0	− 0.5	12.86	13.16	13.45	**13.74**	**14.02**	12.86	13.16	13.45	**13.74**	**14.03**
0.5	− 0.5	12.35	12.64	**12.93**	**13.22**	**13.51**	12.35	12.64	**12.93**	**13.22**	**13.51**
1	− 0.5	11.85	12.13	**12.42**	**12.70**	**12.99**	11.85	12.14	**12.42**	**12.71**	**12.99**
1.5	− 0.5	11.35	**11.63**	**11.92**	**12.20**	**12.48**	11.35	**11.64**	**11.92**	**12.20**	**12,48**
2	− 0.5	**10.86**	**11.14**	**11.42**	**11.70**	**11.98**	**10.86**	**11.14**	**11.42**	**11.70**	**11.98**
0	− 1	12.79	13.08	13.37	**13.66**	**13.95**	12.79	13.08	13.37	**13.67**	**13.96**
0.5	− 1	12.28	12.56	**12.85**	**13.14**	**13.43**	12.28	12.57	**12.86**	**13.14**	**13.43**
1	− 1	11.77	12.06	**12.34**	**12.63**	**12.91**	11.77	12.06	**12.35**	**12.63**	**12.92**
1.5	− 1	11.28	**11.56**	**11.84**	**12.12**	**12.40**	11.28	**11.54**	**11.84**	**12.13**	**12.41**
2	− 1	**10.79**	**11.07**	**11.34**	**11.62**	**11.90**	**10.79**	**11.07**	**11.35**	**11.63**	**11.91**
0	− 1.5	12.71	13.00	13.29	**13.58**	**13.87**	12.71	13.00	**13.30**	**13.59**	**13.88**
0.5	− 1.5	12.20	12.49	**12.77**	**13.06**	**13.35**	12.20	12.49	**12.78**	**13.07**	**13.35**
1	− 1.5	11.70	**11.98**	**12.26**	**12.55**	**12.83**	11.70	**11.99**	**12.27**	**12.56**	**12.84**
1.5	− 1.5	11.20	**11.48**	**11.76**	**12.04**	**12.32**	11.20	**11.49**	**11.77**	**12.05**	**12.33**
2	− 1.5	**10.72**	**10.99**	**11.27**	**11.54**	**11.82**	**10.72**	**11.00**	**11.28**	**11.56**	**11.83**
0	− 2	12.64	12.92	13.21	**13.50**	**13.79**	12.64	12.93	**13.22**	**13.51**	**13.80**
0.5	− 2	12.13	12.41	**12.70**	**12.98**	**13.26**	12.13	12.42	**12.70**	**12.99**	**13.28**
1	− 2	11.62	**11.91**	**12.19**	**12.47**	**12.75**	11.62	**11.91**	**12.20**	**12.48**	**12.76**
1.5	− 2	11.13	**11.41**	**11.68**	**11.96**	**12.24**	11.13	**11.41**	**11.70**	**11.98**	**12.26**
2	− 2	**10.64**	**10.92**	**11.19**	**11.46**	**11.74**	**10.64**	**10.92**	**11.20**	**11.48**	**11.76**

The opportunity cost of capital of the selling firm (buying firm), ${k}_{s}\left({k}_{B}\right)$, is fixed at 11% p.a., while that of the buying ones (selling ones), ${k}_{B}({k}_{S})$, varies between 11 to 15% p.a. All rates ${(m}_{y}^{*})$ are expressed in percentages per annum. The numbers highlighted in bold are solutions yielding ${m}_{y}^{*}<{k}_{B} ({m}_{y}^{*}<{k}_{S})$. The exogenous parameters assumed here are $\omega =97\%; v=75\%; \tau =1; n=90$ days; and $\gamma =30$

#### Robustness check: relaxing the assumption of ${\varvec{\gamma}}$

Our numerical simulation initially assumes the value of $\gamma =30$, which links the TC-Murabaha rate ($m$) to the proportion $\theta$ of buyers who buy the goods on the spot (see Eq. (7)). Our results are robust to even significant reductions in $\gamma$. Table 4 illustrates the optimum TC-Murabaha rate under various demand and supply elasticities levels when $\gamma$ equals $10$ and $20$ in Panels 1 and 2, respectively. With the lower values of $\gamma$, the numbers of ${m}_{y}^{*}$ that are higher than the opportunity cost, $k$, (${m}_{y}^{*}>k$) drop to only one case, in contrast to 4 cases in our base case numerical simulation illustrated in Table 1. Therefore, reducing the assumption of $\gamma$ improves our results by lowering the optimum TC-Murabaha rate to the values depicted in Table 1. This is consistent with our proposition.

**Table 4:** Robustness checks for optimum TC-Murabaha rates with different $\gamma$ values

${\epsilon }_{S}$	${\epsilon }_{B}$									
	0		− 0.5		− 1		− 1.5		− 2	
	${\alpha }_{y}^{*}$	${m}_{y}^{*}$	${\alpha }_{y}^{*}$	${m}_{y}^{*}$	${\alpha }_{y}^{*}$	${m}_{y}^{*}$	${\alpha }_{y}^{*}$	${m}_{y}^{*}$	${\alpha }_{y}^{*}$	${m}_{y}^{*}$
*Panel A:*$\gamma =10$										
2	8.95	9.82	8.66	9.48	8.37	9.13	8.07	8.78	7.78	8.44
1.5	10.00	11.11	9.71	10.75	9.42	10.39	9.12	10.04	8.83	9.69
1	11.08	12.45	10.78	12.08	10.49	11.71	10.19	11.35	9.90	10.98
0.5	12.17	13.85	11.87	13.47	11.57	13.09	11.27	12.71	10.98	12.33
0	13.28	**15.31**	12.97	14.91	12.67	14.51	12.37	14.12	12.07	13.73
*Panel B:*$\gamma =20$										
2	10.97	12.32	10.85	12.17	10.73	12.02	10.61	11.86	10.49	11.71
1.5	11.54	13.04	11.42	12.89	11.30	12.73	11.17	12.58	11.05	12.43
1	12.11	13.78	11.99	13.62	11.87	13.47	11.75	13.31	11.63	13.16
0.5	12.69	14.54	12.57	14.38	12.45	14.22	12.33	14.06	12.20	13.90
0	13.28	**15.31**	13.15	**15.14**	13.03	14.98	12.91	14.82	12.79	14.66

The parameter ${\epsilon }_{B}$ denotes price elasticity of demand, while ${\epsilon }_{S}$ represents the price elasticity of supply. Furthermore, ${\alpha }_{y}^{*}$ is annualized optimum TC rate, i.e., ${\alpha }_{y}^{*}=[(1+{\alpha }^{*}{)}^\frac{360}{n}-1]$, while ${m}_{y}^{*}$ represents annualized optimum TC-Murabaha rate, where ${m}_{y}^{*}={\alpha }_{y}^{*}/(1-{\alpha }_{y}^{*})$. All rates ${(\alpha }_{y}^{*}, {m}_{y}^{*})$ are expressed in percentages per annum. The numbers highlighted in the bold font are solutions yielding ${m}_{y}^{*}>k$. The exogenous parameters assumed here are: $\omega =97\%;v=75\%; {k}_{S}={k}_{B}=k=15\%; \tau =1;$ and $n=90$ days

Furthermore, our results also suggest that elasticities play a significant role in explaining the variability of ${m}_{y}^{*}$ under lower values of $\gamma$. This can be inferred from the increase in spreads between the highest and the lowest value of ${m}_{y}^{*}$ in our simulation with lower $\gamma$ values.^42^ These spreads between the two ${m}_{y}^{*}$ for $\gamma$ values of $30$, $20,$ and $10$, respectively, are $2.44\%$, $3.60\%$, and $6.87\%$. Thus, a lower level of $\gamma$ selected in our numerical simulation yields more robust results. This is because a higher value of the discount-demand parameter $\gamma$ weakens the effect of ${\epsilon }_{S}$ and ${\epsilon }_{B}$ on ${m}_{y}^{*}$, thereby yielding a higher possibility of ${m}_{y}^{*}>k$.

In conclusion, our numerical implementation generates optimum TC-Murabaha rates that are a function of not only short-run costs of capital but also product price elasticities and the other parameters. That is, ${m}^{*}=f({k}_{S},\hspace{0.17em}{k}_{B},\hspace{0.17em}{\epsilon }_{S},\hspace{0.17em}{\epsilon }_{B},\hspace{0.17em}v,\hspace{0.17em}\tau ,\hspace{0.17em}\omega ,\hspace{0.17em}\gamma )$. Our numerical experimentations show the potential to offer TC-Murabaha rates below the opportunity cost of capital of the selling firm*.* This supports the argument that vendor financing (or TC-Murabaha) is superior to a bank loan (or contemporary Murabaha). This is consistent with the fact that the TC-Murabaha can provide a more stable credit rate over time than a lending facility with varying interest rates (see Ng et al. 1999). As a result, constrained buyers shift to vendor financing, particularly in periods of monetary contractions, when banks may not offer credit with competitive rates.

#### Robustness check: relaxing the assumption of ${\varvec{\omega}}$

Throughout this paper, we have assumed the repayment rate ($\omega$) to be 97%. This parameter controls the likelihood of buyers defaulting on the repayment of the TC-Murabaha facility. In this subsection, we relax this assumption to assess the impact of default on how the supplier sets the discount rates. Our simulation results are provided in Table 5.

**Table 5:** Robustness checks for optimum TC-Murabaha rates with different $\omega$ values

$\omega$(%)	${\epsilon }_{S}$	${\epsilon }_{B}$									
		0		− 0.5		− 1		− 1.5		− 2	
		${\alpha }_{y}^{*}$	${m}_{y}^{*}$	${\alpha }_{y}^{*}$	${m}_{y}^{*}$	${\alpha }_{y}^{*}$	${m}_{y}^{*}$	${\alpha }_{y}^{*}$	${m}_{y}^{*}$	${\alpha }_{y}^{*}$	${m}_{y}^{*}$
100	2	5.46	5.78	5.42	5.73	5.37	5.68	5.33	5.63	5.28	5.57
	1.5	5.85	6.22	5.81	6.17	5.76	6.12	5.72	6.07	5.67	6.01
	1	6.25	6.67	6.2	6.61	6.16	6.56	6.11	6.51	6.07	6.46
	0.5	6.65	7.12	6.6	7.07	6.56	7.02	6.51	6.96	6.47	6.91
	0	7.05	7.58	7	7.53	6.96	7.48	6.91	7.42	6.86	7.37
97	2	11.65	13.19	11.59	13.11	11.53	13.03	11.47	12.95	11.41	12.87
	1.5	12.06	13.71	11.99	13.63	11.93	13.55	11.87	13.47	11.81	13.39
	1	12.46	14.23	12.4	14.15	12.34	14.07	12.27	13.99	12.21	13.91
	0.5	12.87	14.77	12.8	14.68	12.74	14.6	12.68	14.52	12.62	14.44
	0	13.28	15.31	13.21	15.22	13.15	15.14	13.09	15.06	13.02	14.97
96	2	13.76	15.95	13.69	15.87	13.63	15.78	13.57	15.7	13.51	15.61
	1.5	**14.01**	**16.30**	**14.01**	**16.30**	**14.01**	**16.30**	13.98	16.25	13.91	16.16
	1	**14.01**	**16.30**	**14.01**	**16.30**	**14.01**	**16.30**	**14.01**	**16.30**	**14.01**	**16.30**
	0.5	**14.01**	**16.30**	**14.01**	**16.30**	**14.01**	**16.30**	**14.01**	**16.30**	**14.01**	**16.30**
	0	**14.01**	**16.30**	**14.01**	**16.30**	**14.01**	**16.30**	**14.01**	**16.30**	**14.01**	**16.30**
75	2	**14.01**	**16.30**	**14.01**	**16.30**	**14.01**	**16.30**	**14.01**	**16.30**	**14.01**	**16.30**
	1.5	**14.01**	**16.30**	**14.01**	**16.30**	**14.01**	**16.30**	**14.01**	**16.30**	**14.01**	**16.30**
	1	**14.01**	**16.30**	**14.01**	**16.30**	**14.01**	**16.30**	**14.01**	**16.30**	**14.01**	**16.30**
	0.5	**14.01**	**16.30**	**14.01**	**16.30**	**14.01**	**16.30**	**14.01**	**16.30**	**14.01**	**16.30**
	0	**14.01**	**16.30**	**14.01**	**16.30**	**14.01**	**16.30**	**14.01**	**16.30**	**14.01**	**16.30**

The parameter ${\epsilon }_{B}$ denotes price elasticity of demand, while ${\epsilon }_{S}$ represents the price elasticity of supply. Furthermore, ${\mathrm{m}}_{\mathrm{y}}^{*}$ ${\mathrm{\alpha }}_{\mathrm{y}}^{*}$ is annualized optimum TC rate, i.e.,${\alpha }_{y}^{*}=[(1+{\alpha }^{*}{)}^\frac{360}{n}-1]$, while ${m}_{y}^{*}$ represents annualized optimum TC-Murabaha rate, where ${m}_{y}^{*}={\alpha }_{y}^{*}/(1-{\alpha }_{y}^{*})$. All rates ${(\alpha }_{y}^{*}, {m}_{y}^{*})$ are expressed in percentages per annum. The numbers highlighted in the bold font are corner solutions implied by ${\alpha }^{*}=\overline{\alpha }={\gamma }^{-1}$. The exogenous parameters assumed here are $\gamma =30$;$v=75\%$; ${k}_{S}={k}_{B}=15\%$;$\tau =1$; and n = 90 days. $\omega$ is the percentage of TC paid in one lump sum

When the repayment rate increases, the supplier will entice the customer to use TC by setting lower cash discount rates. In the extreme case, when all the buyers are sure to repay ($\omega$ = 100%), the optimum TC-Murabaha discount rate (${m}_{y}^{*}$) will be very low, in the region of only 5%-7%. This result is consistent across the broad spectrum of elasticities. Discounts are lowest for inelastic demand and supply and highest otherwise. We have already observed a qualitatively similar effect for the base case. When $\omega$ = 97%, discounts increase only marginally, i.e., in the range of 11%-15%, and are also lowest at the inelastic end.

When $\omega$ decreases, the risk of default increases. This leads to buyer quality deterioration, forcing the supplier to incentivize more customers to pay cash. That is, to take less TC. This is done to offset the increased risk of default. As the TC default risk increases, we obtain a corner solution with $\overline{\alpha }={\gamma }^{-1}$ (see Eq. 8). When this “corner” is reached, the supplier stops offering any TC and offers the largest cash discount possible at a level high enough to convince all customers to shift to cash purchase. At this level of cash discount, the proportion of cash customers becomes $\theta =1$ (see again Eq. 8). Consequently, no customer chooses TC-Murabaha, i.e., $(\theta -1=0)$. This is illustrated in Table 5 for $\omega =96\%$ and confirmed for $\omega =75\%$. In the latter case, any combination of elasticities results in the abandonment of TC as the default risk is high.

The case where $\omega =96\%$ is a transition stage where some combinations of low elasticities still result in TC arrangements, as illustrated in Table 5. Here too, the higher the combined elasticities, the higher is the cash discount. However, at some values of elasticities, the discount becomes constant as the supplier eliminates the uncertainty by inducing all customers to pay cash, thereby helping them do so at the price of a very enticing cash discount ${(\alpha }_{y}^{*}=14.01\%, {m}_{y}^{*}=16.30\%)$.

#### Deliberating on our general results

Our general results contradict Cuñat (2007) and Klapper et al. (2012). They argue that TC has a higher implicit interest rate than bank credits because suppliers require insurance and default premia to remunerate borrowers’ financial constraints. This is not the case for our analysis. Conversely, suppliers benefit from better collateral asset liquidation, information acquisition, and demand risk-sharing. This result is consistent with Nilsen (2002), Ge and Qiu (2007), Fabbri and Menichini (2010), and Sautner and Vladimirov (2018). Apart from this, the advantage of TC-Murabaha is observed in the real world with market imperfections. That is, where there is endemic asymmetric information and the borrowing rate is higher than the lending rate. Our modeling, however, also confers advantages to the selling firm even when they operate in an efficient financial system through price discrimination involving supply and demand price elasticities. In this respect, our results corroborate with Brennan et al. (1988).

### Insinuating the implications of our results on the financial architecture

**Fig. 8:**
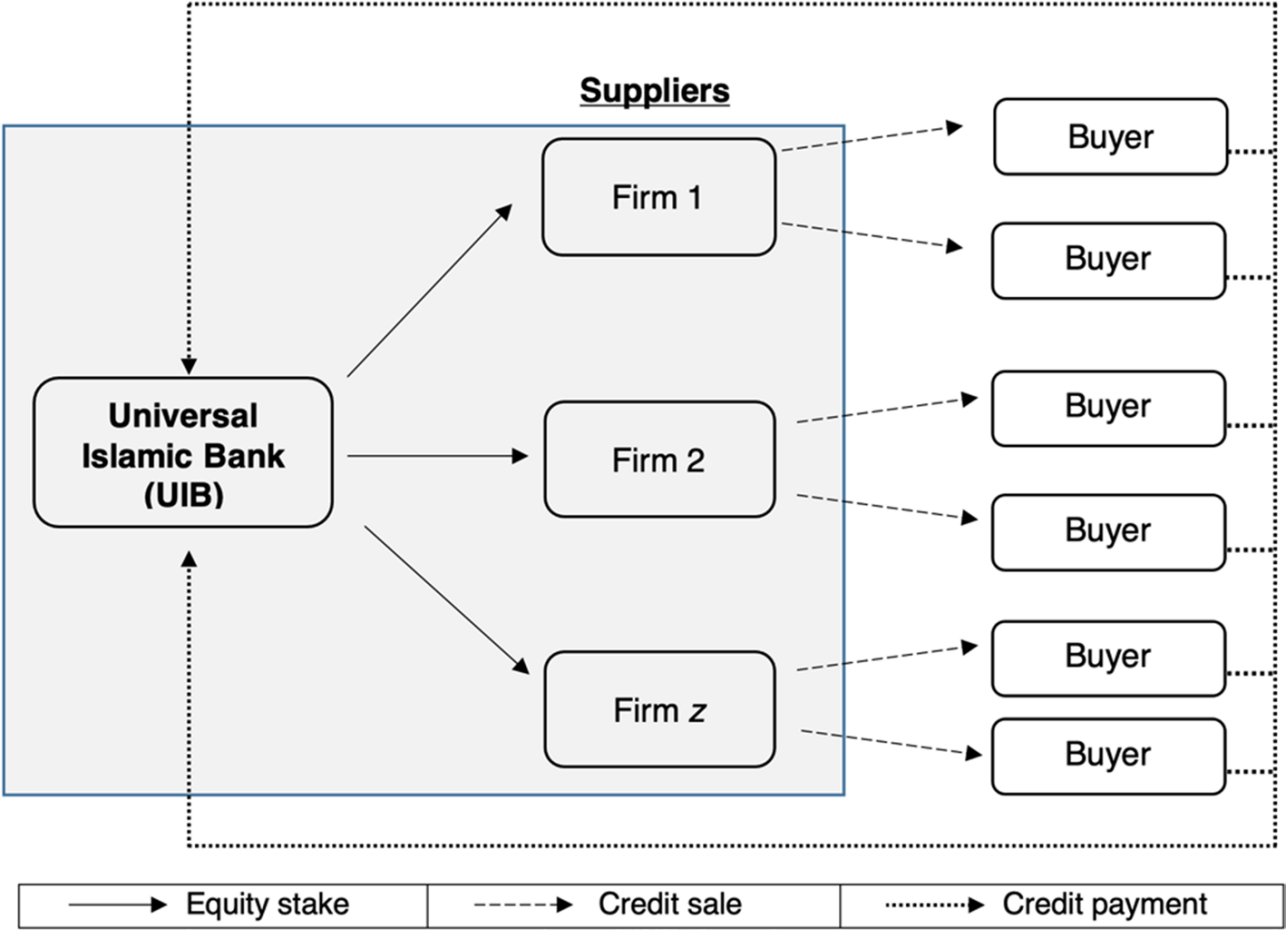
Universal Islamic banking architecture. *Note*: In countries that do not allow universal banking, the UIB above can be substituted by a Global Markets and Asset Management of a Financial Conglomerate

Fundamentally, our findings imply the employment of the universal Islamic banking architecture (see Fig. 8) instead of the contemporary Islamic commercial banking one to avail of the advantages of TC and to conform to the objectives of the Islamic law.^43^ This is due to the following reasons. First, the Universal Islamic Bank (UIB) architecture allows a financial intermediary to own an equity or a quasi-equity (in the form of a participating preferred lease) stake in a selling firm (i.e., Firms_1-*Z*_).^44^ This is consistent with the spirit of risk-sharing in Islam and conveys resilience to the financial system (see Ebrahim et al. 2017). The UIB’s investment in the selling firms is akin to the medieval profit and loss form of financing.

Second, the selling firm owned by the UIB can sell its products (or services) on credit, employing the TC-Murabaha facility to buying firms in the *real* sector of the economy. It remedies the chronic ownership and the fragile long-term debt of contemporary Murabaha. The former is easily tackled as the UIB no longer needs to pre-own the goods ‘sold’—they are pre-owned by its inter-linked selling firms.^45^ The latter is solved by substituting the risk-sharing mechanism of TC in the *real* sector of the economy (see again Sect. 3.2).

Third, a UIB serves a crucial role as a receivable collector. While a selling firm is better at evaluating and enforcing the contract (Petersen and Rajan 1997; Cuñat 2007), a UIB is better at collecting account receivables as it has a more established network system. Thus, the UIB architecture has the potential to alleviate the cost of payment collection in the TC finance as expressed by Biais and Gollier (1997).

Although the above intuitive model is theoretically appealing, a number of conditions need to be addressed to implement it in the real world. The main challenge comes from the regulatory constraints^46^ in some countries that would subject the overall holding company of the universal bank to capital adequacy, leverage ratio, and liquidity requirements.^47^

Nonetheless, the proposed model best fits within a modified universal banking system of Germany where the banks are permitted to have an equity stake in addition to loans, with voting rights and even placing their representative on the board of directors of the firms they serve (Boyd et al. 1998; Guinnane 2002; Neuhann and Saidi 2018).^48^ The model may also be implemented within an altered Japanese main banking system, which resembles Germany’s. This is despite the post-WWII regulation, which does not permit Japanese banks to have an equity position in non-bank firms of more than 5%, akin to that of the USA. In emulating the German financial system, Japan’s financial architecture resembles the *keiretsu* system, where companies are related to each other and the main bank by mutual shareholding (Benston 1994; Miwa and Ramseyer 2002; Sueyoshi et al. 2010). This structure endows universal banks (i) with economies of scale and scope, (ii) to actively promote corporate governance, and (iii) to be resilient to financial distress (Berglöf and Perotti 1994; Berlin et al. 1996; Gorton and Schmid 2000). Thus, we envision an improved version of Germany and Japan’s universal banking models to be more appropriate to accommodate the proposed Islamic banking architecture.

In the extreme case, where national regulations do not allow a universal banking model, our proposal can still be implemented within a financial conglomerate structure where the Global Markets and Asset Management Divisions of the Islamic financial services company own an equity (or quasi-equity) stake in selling firms. Our assertions are confirmed in the specialized banking system such as that of the USA, where the Gramm-Leach-Bliley (GLB or the Financial Modernization) Act of 1999 allows the consolidation of commercial banks, investment banks, securities firms, and insurance companies (Broome and Markham 2005). This Act has created financial behemoths of the likes of Bank of America Corporation, Citigroup Inc., Goldman Sachs Group Inc., JP Morgan Chase and Company, Morgan Stanley Inc., etc. The USA is not an isolated case. Other countries, too, allow financial conglomerates. This includes Australia, Canada, France, Italy, the United Kingdom, etc.

## Conclusion

This paper proposes to economically revitalize Islamic banking by replacing the controversial Murabaha facility with a TC facility. Banking Murabaha has been the backbone of IBs, accounting for more than 60% of the industry’s mode of financing worldwide. This is why solving the embedded issues of banking Murabaha has the potential to ‘rejuvenate’ Islamic banking in the context of the Pareto principle. This issue is crucial in the post-coronavirus environment as an increase in long-term debt of businesses can slow down the economic recovery as loan obligations force firms to defer expanding their respective businesses. TC illustrates the collaborative efforts of businesses to help each other on a short-term basis. It is cyclical based on the phase of the economy and alleviates both agency costs of debt (i.e., risk-shifting and underinvestment). Moreover, TC can redeem the ill-suited Murabaha financing offered by IBs in the *financial* sector of the economy. We recommend an efficient financial intermediation system in line with the objectives of Islamic law.

We advance the Rashid and Mitra (1999) TC framework in a working capital setting of businesses. This facilitates factoring of accounts receivables. It also revives the spirit of the classic Murabaha facility within the *real* sector as opposed to the banking Murabaha conducted in the *financial* sector of the economy. We then price the credit sale facility by extending the TC literature (Shenoy and Williams 2017) and numerically simulate the same. Our parsimonious model illustrates that the classic Murabaha (in a TC framework) can offer more competitive rates than the banking Murabaha. This is consistent with prominent literature such as Nilsen (2002), Ge and Qiu (2007), Fabbri and Menichini (2010), Klapper and Randall (2011), Chod (2017), and Sautner and Vladimirov (2018).

Our study offers policy implications too. For example, it goes beyond simply adopting the TC concept into the classic Murabaha financing by reconstruing the architecture of Islamic banking into universal banking to offer economies of scale and scope without technically modeling the banking sector (Guinnane 2002; Miwa and Ramseyer 2002; Santos and Rumble 2006; Sueyoshi et al. 2010; Neuhann and Saidi 2018).^49^ Combining TC-Murabaha with universal banking can address the transfer of ownership problems and disengage the facility’s pricing from the market rates. In addition, it facilitates optimum supply chain management and active corporate governance of the related firm, thereby alleviating financial fragility as illustrated in Berglöf and Perotti (1994), Berlin et al. (1996), Gorton and Schmid (2000), and Chod (2017). This is especially true in countries where the regulations impose barriers on banking, securities, and insurance businesses, the next best alternative is a financial conglomerate structure emulating Goldman Sachs Group Inc. in the USA.

We believe our results will stimulate a rethink of contemporary Islamic banking, thereby increasing its efficiency. We argue that such a reassessment will enable it to dissipate systemic risk better and thus make the financial system more resilient to shocks. This will further the expansion of businesses worldwide, enhancing global growth.

## Permissions

Figures 2 and 3 (pages 53 and 54) are reproduced/sourced from Wojakowski et al. (2019), as noted in the manuscript. Wojakowski and Ebrahim are also the co-authors of this submitted manuscript. Financial Markets, Institutions, and Instruments (published by Wiley – online and print) allow authors to reuse their article in a new publication of which they are the authors. (https://onlinelibrary.wiley.com/page/journal/14680416/homepage/permissions.html).

## Data Availability

The study used only simulated data.

## Appendix A: Linking the Classic Murabaha Mark-up ($m$) with the Trade Credit (TC) Discount ($\alpha$)

Buyers can either pay the discounted price $p=\left(1-\alpha \right)P$ immediately, or they can pay $P$ in $n$ days. Therefore

$$P=\frac{p}{1-\alpha }\hspace{0.33em}.$$

On the other hand, if $p$ is the current price in the classic Murabaha mark-up terminology, then $P=p(1+m)$, where $m$ is the mark-up rate. It follows that

43 $$\begin{array}{ccc}\frac{p}{1-\alpha }& =& p\left(1+m\right)\iff 1-\alpha =\frac{1}{1+m}\\ & \iff & \alpha =1-\frac{1}{1+m}=\frac{m}{m+1}\\ & \iff & m=\frac{1}{1-\alpha }-1=\frac{\alpha }{1-\alpha }\hspace{0.33em}.\end{array}$$

The above equation thus integrates the classic Murabaha mark-up $\left(m\right)$ with the trade discount ($\alpha$).

## Footnotes

1 All the banks in Iran and Sudan are Islamic, while more than half of the banking assets in Saudi Arabia and Brunei Darussalam are also Islamic. The proportion of assets of IBs in Kuwait, Malaysia, Qatar, Djibouti, Bangladesh, UAE, Jordan, Palestine, Pakistan, Bahrain, and Oman range between 15 and 50% (see IFSB 2021 for details on IBs’ assets in other countries of the world).

2 The ‘classic’ Murabaha transaction in the medieval period of Islam is akin to a fiduciary forward (i.e., mark-up) sale at a stated profit margin. This is organized in the *real* (i.e., business) sector of the economy. On the contrary, ‘banking’ Murabaha is conducted in the *financial* sector, where IBs act as intermediaries. IBs purchase the intermediate or final goods ordered by their clients (debtors) from the market and then sell them on credit to the clients after the ownership of the assets is transferred to the banks. In practice, however, the actual transfer of ownership from the seller to the bank to the debtor hardly occurs. Instead, IB delegates the debtors to buy the pertinent goods themselves using the agency contract.

3 Kuran (2018, p. 1308) states the following: “….many of the financial procedures touted as Islamic are ruses that medieval jurists developed expressly to circumvent inconvenient legal barriers. Certain ruses such as the double sale used to bury interest payment in a fictitious transaction, have Christian equivalents dating from the Middle Ages, when the Church prohibited interest.”

4 A resilient risk-sharing contract such as a trade credit facility is a loan in which a genuine ‘seller’ of a tangible good (or services) in the *real* sector of the economy has a quasi-equity interest in the ‘buying’ firm. In the good state of the economy, the seller is able to make a decent profit, while in the poor state, it would bear the loss of goods sold by recouping it from the buyer, refurbishing it, and then reselling the same.

5 Systemic risk is defined as the risk of severance in the flow of financial services that is (i) induced by the deterioration of all (or parts) of the financial system; and (ii) has the potential to negatively impact on the businesses in the *real* sector of the economy (Acemoglu et al. 2015; Grundke 2019).

6 The interconnectedness of the global financial system is well documented (see Raddant and Kenett 2021) and IBs are no exception. They have become more integrated with the global financial system as they grew in size and operational jurisdictions (Karim and Naeem 2022). While high cross-border financial integration brings about efficiency, it can also increase the incidence of the banking crisis and escalate contagion to other territories (Inekwe and Valenzuela 2020). Previous studies (Čihák and Hesse 2010; Abedifar et al. 2013) document that the systematic risk of IBs increases as they become larger. The relatively smaller size and lesser exposure to other banks and derivatives kept IBs more resilient during the 2008 Global Financial Crisis (Čihák and Hesse 2010; Abedifar et al. 2013). However, since then, the Islamic banking industry has experienced exponential growth in its market share and connection with the global financial system (IFSB 2021). It is thus plausible that IBs can “transmit systemic risks” to other sectors or territories (Chakroun and Gallali 2017). Some examples of IBs’ collapses and distress across the world include Denmark’s Islamic International Bank (IIB) in 1985 and 1986 (Grais and Pellegrini 2006), the Islamic Bank Ltd (IBL) of South Africa in 1997 (Nathie 2010), Ihlas Finance House (IFH) of Turkey in 2001 (Ali 2007), and Muamalat Bank of Indonesia in 2015 (Maulia 2019).

7 It should be noted that the primary objective of our paper is to propose the replacement of the banking Murabaha facility by incorporating TC in the Islamic banking architecture. Accordingly, we do not model the banking sector as it is beyond the mandate of our paper. At best, we insinuate on how a truly Islamic banking architecture should be in Sect. 4.3.

8 This rationale ensues from Coase (1937) and Alchian (1950), who hypothesize that businesses evolve to the form that helps them survive the competitive environment by delivering products and services demanded by their customers at the lowest prices by mitigating transaction costs (including financing costs).

9 The sheer size of Amazon and Walmart helps them to get better credit terms from their suppliers. A Universal Islamic Bank (UIB) that is envisioned in this paper (and described in Sect. 4.3) would also be a gigantic firm with vast resources and would also be able to get better terms of credit from their stakeholder firms.

10 The *Qur’ānic* verse (2:275) segregates trade-based financing (in the *real* sector of the economy) from debt financing (in the *financial* sector). It encourages the employment of the former while admonishing the use of the latter.

11 The objectives of Islamic law (*Maqāsid Al-Sharī’ah*) are to ensure the preservation of faith, intellect, life, lineage, and wealth of human beings (see Kamali 2000). They were developed by several jurists in the medieval ages to deeply comprehend the meaning of the Qur’ān and the traditions of Prophet Muhammad (Kamali 2000). This subject is considered to be the most important intellectual means for reform and renewal of the Muslim world. This is because it can induce fresh ideas dynamically. The jurists stressed on treating economic agents equitably (*Adl*) and on curbing harm (*Darar*). These two issues have ethical connotation as they impact on the welfare (*Maslaha*) of economic agents.

12 The reason for using numerical simulation in lieu of empirical analysis is because a framework of Islamic banking with TC does not currently exist. Theoretical conceptualization accompanied with numerical simulation is the best way of testing our proposed framework and advancing Islamic banking.

13 Our proposed Murabaha is derived from the TC framework. The previous literature documents the countercyclicality of TC (e.g., Nilsen 2002; Carbo-Valverde et al. 2016). To confirm this in our proposed Murabaha, we perform a numerical simulation extending the R&M model in Sect. 4.2. R&M model TC in a partial equilibrium setting by employing an optimizing model. That is, they analyze a firm (i.e., business) selling goods to a buyer in the *real* sector of the economy. They incorrectly term this as a demand-side one instead of a supply-side one. Their results are flawed as they use negative elasticities in their numerical simulations. In contrast, we model the supply chain comprising of a selling firm and multiple buying ones, thereby incorporating the respective supply as well as the demand-side. We also incorporate positive elasticity of supply for the selling firm and negative elasticity of demand for the buying ones. This makes our analysis quite rich in integrating the conflict of interest between the two competing economic agents.

14 *Keiretsu* is defined by Santos and Rumble (2006, p. 424) as “*groups of Japanese firms* [businesses] *from the nonfinancial and financial sectors that are connected by interlocking shareholdings*.”

15 The focus of our paper is not on regulations. We therefore implicitly assume that the proposed universal Islamic banking architecture (or a financial conglomerate) adheres to the local, national and international regulations such as the Basel III accord. See Bitar et al. (2021) for more information on this issue.

16 In countries with the specialized banking system, a financial conglomerate structure akin to that of Goldman Sachs is the next best alternative to the universal banking architecture.

17 The religious scholars impose a precondition on the asset reputedly being sold on credit. It should not constitute a medium of exchange in terms of being a currency or a commodity in medieval Islam (Al-Zuhayli 2003).

18 The basic tenets of Islam reinforce helping the poor and the underprivileged. This includes facilitating access to them for financial services thereby leading to their financial inclusion (see Salleh et al. 2014). Unfortunately, contemporary IBs financially exclude the poor and the underprivileged.

19 This injunction is akin to that highlighted in Deuteronomy (23: 19–20), Exodus (22: 25), and Leviticus (25: 36–37).

20 According to Bloomberg, the Islamic bond issuance average annual growth rate was 68.38% compared to 13.03% in its conventional counterpart, between 1995 and 2020. The Islamic bond issuance even increased by 41.66% in 2019 when the issuance of conventional bonds decreased by 0.06%.

21 Ebrahim and Sheikh (2018) allege the static nature of Islamic rulings as one of the causes of why Muslim-majority countries lack efficient financial intermediaries and thus tend to be economically underdeveloped in contrast to the rest of the world. These rulings have been static as the deductive basis employed by the Islamic scholars has been from a purely legal perspective instead of the objectives of Islamic law stemming from economic rationale. The legally based rulings are most likely to be conducted through analogical reasoning, restricted to very limited situations. In contrast, the ones based on the economic rationale are yet to be developed. This not only considers the meaning of the text but also contemplates the Lawgiver’s intent in the scriptural source (Kamali 2000). The issue of underdevelopment of Muslim economies has been insinuated earlier by Max Weber (in his well-known book The Protestant Ethic and the Spirit of Capitalism (1904-5/1958)) to its cultural doctrines and the lack of dynamic review of the same over changing intellectual and socio-economic conditions.

22 There is a long-standing debate in the literature on whether TC and bank loans are substitutes or complements (Lau and Schaede 2020). Cuñat (2007, p. 494–495) distinguishes the two as follows: “….*trade credit levels are forward looking because they depend on the future value of the relationship between supplier and customer, where bank credit is backward looking and depends on the collateral accumulated in previous periods..*” Our paper considers the two financing facilities as substitutes. However, when the default rate escalates, as indicated in our Sect. 4.2.5, TC and the risk-sharing quasi-equity one advanced by a UIB (discussed in our Sect. 4.3) become complements.

23 This issue can be elaborated as follows. Consider a firm (i.e., business) buying information technology equipment and hiring technicians, the firm can subcontract these services to another. This expands the scope of TC to intangibles.

24 The TC-Murabaha discount rate ($m$) is linked to that of the TC rate ($\alpha$) as illustrated in Appendix A as follows: $m=\alpha /(1-\alpha )$.

25 We implicitly assume that the intermediate (or final) goods or services sold via TC have a (i) meaningful use; and (ii) commercial value.

26 This Sect. 3.2.1, emulates the R&M analysis on the supply-side, while Sects. 3.2.2 and 3.2.3 extend the same to the demand-side and impose the market-clearing conditions respectively. We advance R&M’s perspective in our numerical simulation Sect. 4.2.

27 Because the specification of $\theta$ and $\omega$ are the same for supply-side as well as for the demand-side, we do not use subscripts $S$ in the discount factor $\alpha$ in this and the next paragraph, where $\omega$ is defined.

28 In contrast to our assumptions, R&M postulate a specification for sales net of discount, $S(\alpha )$, before introducing the corresponding elasticity $\epsilon$. They then incorporate negative elasticities in their numerical simulation. This includes the case $\epsilon <1,$ which would have a positive impact on sales $\partial S(\alpha )/\partial \alpha >0$. This approach is problematic because negative elasticities correspond to elasticities of demand. This renders the R&M analysis of supply-side inconsistent with economic theory.

29 Similar to ${V}_{S}$, the buyers’ present value ${V}_{B}$ is also a function of $P,{k}_{B},\tau ,$ and $n$.

30 We ignore the higher value of $\alpha$ as it is greater than 100% and does not make economic sense.

31 This highlights the concern of bankers who contend that they are not able to offer significantly lower financing rates than the captive finance companies of manufactures as they have been subsidized by their parent firm (see Sen 1998).

32 Our result has implications on the structuring of Islamic financial architecture. This is discussed in Sect. 4.3 below.

33 This risk premium incorporates the deadweight cost of default thereby aggravating the underinvestment issue, highlighted under Sect. 2.1.

34 One might argue that the lower TC-Murabaha rate will invite rational investors to exercise arbitrage opportunities in the *real* sector of the economy. This is not feasible for two reasons. First, the literature on TC illustrates how suppliers can comparatively decipher information on their buyers quickly and at a lower cost than that by financial institutions (see again Smith 1987; Brennan et al. 1988; Ng et al. 1999). This includes information on the capacity of the buyers. They can then ‘ration’ their intermediate goods to the buyer to deter the buyer from becoming a competitor by resorting to arbitrage. Second, buyers would face transaction costs while transferring ownership from themselves to secondary buyers. The fact that TC is predominantly used for procuring intermediate goods for production, where the products are not generic, making the market for them to be inexistent.

35 We thank an anonymous referee for pointing out that issuing long-term debt at record low bond rates can have a very positive effect on the long-term financial health of a business of any size. We would agree with this perspective if the lenders do not incorporate inflationary expectations. Besides, the agency issue of risk-shifting is prevalent in most plain-vanilla debt. This exacerbates financial fragility, systemic risk, and thus financial instability.

36 We employ a more conservative assumption of $\gamma =30$. This contrasts with Hill and Riener (1979) and R&M who respectively assume $\gamma =1$ and $\gamma =10$. Nonetheless, our simulation results are still robust for the lower levels of $\gamma$, as illustrated in Sect. 4.2.4.

37 In our notation, the subscript $y$ represents a calendar year. The derivations of both ${\alpha }_{y}^{*}$ and ${m}_{y}^{*}$ are illustrated in Tables 1 and 4.

38 See again Fig. 5, where most of the optimal mark-up rates (${m}_{y}^{*}$’s) lie below the opportunity cost of capital $k=15$. The latter is represented as a translucent horizontal surface.

39 Assuming that $${k}_{S}={k}_{B}=k$$.

40 This may at first seem counter-intuitive, compared to most textbook illustrations of the economic cycle which tend to represent expansions as peaks and contractions as troughs.

41 Since $\frac{\partial {m}_{y}^{*}}{\delta {k}_{B}}\approx \frac{\partial {m}_{y}^{*}}{\delta {k}_{S}}$, the results for ${m}_{y}^{*}=f({k}_{B}|{k}_{S}=11\%)$ and ${m}_{y}^{*}=f({k}_{S}|{k}_{B}=11\%)$ in Table 3 are virtually identical.

42 The highest ${m}_{y}^{*}$ occurs when ${\epsilon }_{S}={\epsilon }_{B}=0$, while the lowest one occurs when ${\epsilon }_{S}=2$ and ${\epsilon }_{B}=- 2$.

43 This subsection suggests an alternative way of conducting financial intermediation (by replacing Murabaha with a TC facility) through a Universal Islamic Bank (UIB). We do not model the banking sector as it is beyond the scope of our paper. We aim to model a UIB in future research.

44 The equity/quasi-equity link between a UIB and the selling firms distinguishes itself from a purely German or a Japanese financial architecture where banks have debt along with equity stakes in their interlinked firms. Our UIB structure aligns the goals of the main bank with that of the interlinked firms. In contrast, the debt link in the German/Japanese system can aggravate fragility, especially when the agency issues between the two are exacerbated (Xie 2007).

45 Here, the firm need not be selling only tangible goods. It can also be a service provider like an accountant, lawyer, etc. In this case, our UIB also increases the economies of scope.

46 These regulatory constraints would include those levied at the local (such as county/state) or national or global (such as the Basel Standards) levels (see again Bitar et al. 2021). At a national level, many countries ‘ring-fence’ commercial and investment banking, such as the US Glass-Steagall Act (or the banking Act of 1933). At an international level, Basel III puts a higher requirement for the universal bank’s investment activities. Basel III mandates a higher risk-weighted capital to engage in ‘non-banking’ activities, such as capital market investment, to cover market and credit risks.

47 The Universal Islamic Bank (UIB) proposed in our paper can increase its efficiency by tapping into the disruptive technology and innovation employed by the contemporary FinTech industry. These involve Artificial Intelligence (AI), Blockchain, Cloud Computing, and Big Data. That is, the four key areas (or the ABCD) of FinTech. AI is instrumental in voice recognition, natural language processing, computer vision for user-account management, fraud detection, machine learning methods, deep learning networks for anti-money laundering, and credit modeling (Lai et al. 2020). Blockchain technology assists in the clearing and settlement payments, trade finance, and verification of customers and counterparties (Arnold 2017). Cloud computing grants access to shared resources, applications, or storage over the internet. It can enable our UIB to store and process data in remote servers instead of local systems (Misra and Doneria 2018). Finally, Big Data aids the UIB in the: (a) analysis of clients’ income and expenditures; (b) segmentation of its customers’ base; (c) risk assessment and fraud prevention; and (d) providing feedback to management to augment customer loyalty (Hasan et al. 2020). We thank an anonymous referee on prompting us to consider the potential role of FinTech in Islamic Banking.

48 Representation in the board of directors of firms mitigates asymmetric information between a UIB and the firms (Kroszner and Strahan 2001).

49 We aim to extend our study by modeling the banking sector in the future.

## Code availability

N/A.
